# No Statistical-Computational Gap in Spiked Matrix Models with Generative Network Priors †

**DOI:** 10.3390/e23010115

**Published:** 2021-01-16

**Authors:** Jorio Cocola, Paul Hand, Vladislav Voroninski

**Affiliations:** 1 Department of Mathematics, Northeastern University, Boston, MA 02115, USA; 2 Khoury College of Computer Sciences, Northeastern University, Boston, MA 02115, USA; p.hand@northeastern.edu; 3 Helm.ai, Menlo Park, CA 94025, USA; vlad@helm.ai

**Keywords:** spiked matrix models, generative networks, rank-one matrix recovery, statistical-computational gap

## Abstract

We provide a non-asymptotic analysis of the spiked Wishart and Wigner matrix models with a generative neural network prior. Spiked random matrices have the form of a rank-one signal plus noise and have been used as models for high dimensional Principal Component Analysis (PCA), community detection and synchronization over groups. Depending on the prior imposed on the spike, these models can display a statistical-computational gap between the information theoretically optimal reconstruction error that can be achieved with unbounded computational resources and the sub-optimal performances of currently known polynomial time algorithms. These gaps are believed to be fundamental, as in the emblematic case of Sparse PCA. In stark contrast to such cases, we show that there is no statistical-computational gap under a generative network prior, in which the spike lies on the range of a generative neural network. Specifically, we analyze a gradient descent method for minimizing a nonlinear least squares objective over the range of an expansive-Gaussian neural network and show that it can recover in polynomial time an estimate of the underlying spike with a rate-optimal sample complexity and dependence on the noise level.

## 1. Introduction

One of the fundamental problems in statistical inference and signal processing is the estimation of a signal given noisy high dimensional data. A prototypical example is provided by spiked matrix models where a signal $y_{🟉}\in R^{n}$ is to be estimated from a matrix *Y* taking one of the following forms:

- **Spiked Wishart Model** in which $Y\in R^{N\times n}$ is given by:
  (1) $$Y=u\;{y_{🟉}}^{T}+\sigma Z,$$
  where σ>0, $u∼N(0,I_{N})$, $Z_{ij}$ are i.i.d. from N(0,1), and *u* and *Z* are independent;
- **Spiked Wigner Model** in which $Y\in R^{n\times n}$ is given by:
  (2) $$Y=y_{🟉}{y_{🟉}}^{T}+\nu H$$
  where ν>0, $H\in R^{n\times n}$ is drawn from a *Gaussian Orthogonal Ensemble* GOE(n), that is, $H_{ii}∼N\left(0,2/n\right)$ for all 1≤i≤n and $H_{ij}=H_{ji}∼N\left(0,1/n\right)$ for 1≤j<i≤n.

In the last 20 years, spiked random matrices have been extensively studied, as they serve as a mathematical model for many signal recovery problems such as PCA [1,2,3,4], synchronization over graphs [5,6,7] and community detection [8,9,10]. Furthermore, these models are archetypal examples of the trade-off between statistical accuracy and computational efficiency. From a statistical perspective, the objective is to understand how the choice of the prior on $y_{🟉}$ determines the critical signal-to-noise ratio (SNR) and number of measurements above which it becomes information-theoretically possible to estimate the signal. From a computational perspective, the objective is to design efficient algorithms that leverage such prior information. A recent and vast body of literature has shown that depending on the chosen prior, gaps can arise between the minimum SNR required to solve the problem and the one above which known polynomial-time algorithms succeed. An emblematic example is provided the Sparse PCA problem where the signal $y_{🟉}$ in (1) is taken to be *s*-sparse. In this case N=O(slogn) number of samples are sufficient for estimating $y_{🟉}$ [2,4], while the best known efficient algorithms require $N=O(s^{2})$ [3,11,12]. This gap is believed to be fundamental. This “statistical-computational gap” has been observed also for Spiked Wigner models (2) and, in general, for other structured signal recovery problems where the prior imposed has a combinatorial flavor (see the next section and [13,14] for surveys).

Motivated by the recent advances of deep generative networks in learning complex data structures, in this paper we study the spiked random matrix models (1) and (2), where the planted signal $y_{🟉}$ has a *generative network prior.* We assume that a generative neural network $G:R^{k}\to R^{n}$ with k<n, has been trained on a data set of spikes, and the unknown spike $y_{🟉}\in R^{n}$ lies on the range of *G*, that is, we can write $y_{🟉}=G\left(x_{🟉}\right)$ for some $x_{🟉}\in R^{k}$. As a mathematical model for the trained *G*, we consider a network of the form:

(3) $$G\left(x\right)=\mathrm{relu}(W_{d}\ldots \mathrm{relu}\left(W_{2}\mathrm{relu}\left(W_{1}x\right)\right)\ldots ),$$

with weight matrices $W_{i}\in R^{n_{i}\times n_{i-1}}$ and relu(x)=max(x,0) is applied entrywise. We furthermore assume that the network is expansive, that is, $n=n_{d}>n_{d-1}>\cdots >n_{0}=k$, and the weights have Gaussian entries. These modeling assumptions and their variants were used in [15,16,17,18,19,20].

Enforcing generative network priors has led to substantially fewer measurements needed for signal recovery than with traditional sparsity priors for a variety of signal recovery problems [17,21,22]. In the case of phase retrieval, [17,23] have shown that under the generative prior (3), efficient compressive phase retrieval is possible with sample complexity proportional (up to log factors) to the underlying signal dimensionality *k*. In contrast, for a sparsity-based prior, the best known polynomial time algorithms (convex methods [24,25,26], iterative thresholding [27,28,29], etc.) require a sample complexity proportional to the square of the sparsity level for stable recovery. Given that generative priors lead to no computational-statistical gap with compressive phase retrieval, one might anticipate that they will close other computational-statistical gaps as well. Indeed, [30] analyzed the spiked models (1) and (2) under a generative network prior similar to (3) and observed no computational-statistical gap in the asymptotic limit k,n,N→∞ with n/k=O(1) and N/n=O(1). For more details on this work and on the comparison of sparsity and generative priors, see Section 2.2.

### Our Contribution

In this paper we analyze the spiked matrix models (1) and (2) under a generative network prior in the nonasymptotic, finite data regime. We consider a *d*-layer feedforward generative network $G:R^{k}\to R^{n}$ with architecture (3). We furthermore assume that the planted spike $y_{🟉}\in R^{n}$ lies on the range of *G*, that is, there exists a latent vector $x_{🟉}\in R^{k}$ such that $y_{🟉}=G\left(x_{🟉}\right)$.

To estimate $y_{🟉}$, we first find an estimate $\hat{x}$ of the latent vector $x_{🟉}$ and then use $G(\hat{x})$ to estimate $y_{🟉}$. We thus consider the following minimization problem (under the conditions on the generative network specified below, it was shown in [15] that *G* is invertible and there exists a unique $x_{🟉}$ that satisfies $y_{🟉}=G\left(x\right)$:

(4) $$\min_{x\in R^{k}}f\left(x\right):=\frac{1}{4}{\left\|G\left(x\right)G{\left(x\right)}^{T}-M\right\|}_{F}^{2},$$

where:

- for the **Wishart model** (1) we take $M=\Sigma_{N}-\sigma^{2}I_{n}$ with $\Sigma_{N}=Y^{T}Y/N$
- for the **Wigner model** (2) we take M=Y.

Despite the non-convexity and non-smoothness of the problem, our preliminary work in [31] shows that when the generative network *G* is expansive and has Gaussian weights, (4) enjoys a favorable optimization geometry. Specifically, every nonzero point outside two small neighborhoods around $x_{🟉}$ and a negative multiple of it, has a descent direction which is given a.e. by the gradient of *f*. Furthermore, in [31] it is shown that the the global minimum of *f* lies in the neighborhoods around $x_{🟉}$ and has optimal reconstruction error. This result suggests that a first order optimization algorithm can succeed in efficiently solving (4), and no statistical-computational gap is present for the spiked matrix models with a (random) generative network prior in the finite data regime. In the current paper, we prove this conjecture by providing a polynomial-time subgradient method that minimizes the non-convex problem (4) and obtains information-theoretically optimal error rates.

Our main contribution can be summarized as follows. We analyze a subgradient method (Algorithm 1) for the minimization of (4) and show that after a polynomial number of steps $\tilde{T}$ and up to polynomials factors in the depth *d* of the network, the iterate $x_{\tilde{T}}$ satisfies the following reconstruction errors:

- in the **Spiked Wishart Model**:
  (5) $$\|G\left(x_{\tilde{T}}\right)-y_{🟉}{\|}_{2}≲\left(1+\frac{\sigma^{2}}{\|y_{🟉}{\|}_{2}^{2}}\right)\sqrt{\frac{k\log(n)}{N}}{\left\|y_{🟉}\right\|}_{2}$$
  in the regime N≳klog(n);
- in the **Spiked Wigner Model**:
  (6) $$\|G\left(x_{\tilde{T}}\right)-y_{🟉}{\|}_{2}≲\frac{\nu }{\|y_{🟉}{\|}_{2}^{2}}\sqrt{\frac{k\log(n)}{n}}{\left\|y_{🟉}\right\|}_{2}.$$

We notice that these bounds are information-theoretically optimal up to the log factors in *n*, and correspond to the best achievable in the case of a *k*-dimensional subspace prior. In particular, they imply that efficient recovery in the Wishart model is possible with a number of samples *N* proportional to the intrinsic dimension of the signal $y_{🟉}$. Similarly, the bound in the Spiked Wigner Model implies that imposing a generative network prior leads to a reduction of the noise by a factor of k/n.
**Algorithm 1:** Subgradient method for the minizimization problem (4)
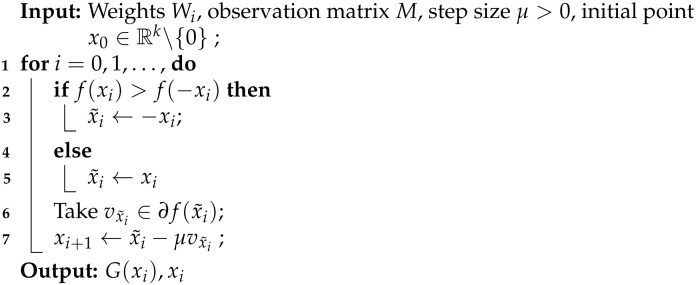


## 2. Related Work

### 2.1. Sparse PCA and Other Computational-Statistical Gaps

A canonical problem in Statistics is finding the directions that explain most of the variance in a given cloud of data, and it is classically solved by Principal Component Analysis. Spiked covariance models were introduced in [1] to study the statistical performance of this algorithm in the high dimensional regime. Under a spiked covariance model it is assumed that the data are of the form:

(7) $$y_{i}=u_{i}y_{🟉}+\sigma z_{i},$$

where σ>0, $u_{i}∼N\left(0,1\right)$ and $z_{i}∼N\left(0,I_{n}\right)$ are independent and identically distributed, and $y_{🟉}$ is the unit-norm planted spike. Each $y_{i}$ is an i.i.d. sample from a centered Gaussian N(0,Σ) with spiked covariance matrix given by $\Sigma =y_{🟉}{y_{🟉}}^{T}+\sigma^{2}I_{n}$, with $y_{🟉}$ being the direction that explains most of the variance. The estimate of $y_{🟉}$ provided by PCA is then given by the leading eigenvector $\hat{y}$ of the empirical covariance matrix $\Sigma_{N}=\frac{1}{N}\sum_{i=1}^{N}y_{i}y_{i}^{T}$, and standard techniques from high dimensional probability can be used to show that (we write f(n)≳g(n) if f(n)≥Cn for some constant C>0 that might depend σ and $\|y_{🟉}{\|}^{2}$. Similarly for f(n)≲g(n) as long as N≳n,

(8) $$\min_{\epsilon =\pm }{\left\|\epsilon \hat{y}-y_{🟉}\right\|}_{2}≲\sqrt{\frac{n}{N}},$$

with overwhelming probability. Note incidentally that the data matrix $Y\in R^{N\times n}$ with rows ${\left\{y_{i}^{T}\right\}}_{i}$ can be written as (1).

Bounds of the form (8), however, become uninformative in modern high dimensional regimes where the ambient dimension of the data *n* is much larger than, or on the order of, the number of samples *N*. Even worse, in the asymptotic regime n/N→c>0 and for $\sigma^{2}$ large enough, the spike $y_{🟉}$ and the estimate $\hat{y}$ become orthogonal [32], and minimax techniques show that no other estimators based solely on the data (7), can achieve better overlap with $y_{🟉}$ [33].

In order to obtain consistent estimates and lower the sample complexity of the problem, therefore, additional prior information on the spike $y_{🟉}$ has to be enforced. For this reason, in recent years various priors have been analyzed such as positivity [34], cone constraints [35] and sparsity [32,36]. In the latter case $y_{🟉}$ is assumed to be *s*-sparse, and it can be shown (e.g., [33]) that for N≳slogn and n≳s, the *s*-sparse largest eigenvector ${\hat{y}}_{s}$ of $\Sigma_{N}$

$${\hat{y}}_{s}=\underset{y\in S_{2}^{n-1},{\left\|y\right\|}_{0}\leq s}{\mathrm{argmax}}y^{T}\Sigma_{N}y$$

Satisfies with high probability the condition:

$$\min_{\epsilon =\pm }{\left\|\epsilon {\hat{y}}_{s}-y_{🟉}\right\|}_{2}≲\sqrt{\frac{s\log n}{N}}.$$

This implies, in particular, that the signal $y_{🟉}$ can be estimated with a number of samples that scales linearly with its intrinsic dimension *s*. These rates are also minimax optimal; see for example [4] for the mean squared error and [2] for the support recovery. Despite these encouraging results, no currently known polynomial time algorithm achieves such optimal error rates and, for example, the covariance thresholding algorithm of [37] requires $N≳s^{2}$ samples in order to obtain exact support recovery or estimation rate

$$\min_{\epsilon =\pm }{\left\|\epsilon {\hat{y}}_{s}-y_{🟉}\right\|}_{2}≲\sqrt{\frac{s^{2}\log n}{N}},$$

as shown in [3]. In summary, only computationally intractable algorithms are known to reach the statistical limit N=Ω(s) for Sparse PCA, while polynomial time methods are only sub-optimal, requiring $N=\Omega (s^{2})$. Notably, [38] provided a reduction of Sparse PCA to the planted clique problem which is conjectured to be computationally hard.

Further strong evidence for the hardness of sparse PCA have been given in a series of recent works [39,40,41,42,43]. Other computational-statistical gaps have also been found and studied in a variety of other contexts such as sparse Gaussian mixture models [44], tensor principal component analysis [45], community detection [46] and synchronization over groups [47]. These works fit in the growing and important body of literature aiming at understanding the trade-offs between statistical accuracy and computational efficiency in statistical inverse problems.

We finally note that many of the above mentioned problems can be phrased as recovery of a spike vector from a spiked random matrix. The difficulty can be viewed as arising from simultaneously imposing low-rankness and additional prior information on the signal (sparsity in case of Sparse PCA). This difficulty can be found in sparse phase retrieval as well. For example, [25] has shown that m=O(slogn) number of quadratic measurements are sufficient to ensure well-posedness of the estimation of an *s*-sparse signal of dimension *n* lifted to a rank-one matrix, while $m\geq O(s^{2}/\log^{2}n)$ measurements are necessary for the success of natural convex relaxations of the problem. Similarly, [48] studied the recovery of simultaneously low-rank and sparse matrices, showing the existence of a gap between what can be achieved with convex and tractable relaxations and nonconvex and intractable methods.

### 2.2. Inverse Problems with Generative Network Priors

Recently, in the wake of successes of deep learning, generative networks have gained popularity as a novel approach for encoding and enforcing priors in signal recovery problems. In one deep-learning-based approach, a dataset of “natural signals” is used to train a generative network in an unsupervised manner. The range of this network defines a low-dimensional set which, if successfully trained, contains or approximately contains, target signals of interest [19,21]. Non-convex optimization methods are then used for recovery by optimizing over the range of the network. We notice that allowing the algorithms the complete knowledge of the generative network architecture and of the learned weights is roughtly analogous to allowing sparsity-based algorithms the knowledge of the basis or frame in which the signal is modeled as sparse.

The use of generative network for signal recovery has been successfully demonstrated in a variety of settings such as compressed sensing [21,49,50], denoising [16,51], blind deconvolution [22], inpainting [52] and many more [53,54,55,56]. In these papers, generative networks significantly outperform sparsity based priors at signal reconstruction in the low-measurement regime. This fundamentally leverages the fact that a natural signal can be represented more concisely by a generative network than by a sparsity prior under an appropriate basis. This characteristic has been observed even in untrained generative networks where the prior information is encoded only in the network architecture and has been used to devise state-of-the-art signal recovery methods [57,58,59].

Parallel to these empirical successes, a recent line of works have investigated theoretical guarantees for various statistical estimation tasks with generative network priors. Following the work of [15,21] gave global guarantees for compressed sensing, followed then by many others for various inverse problems [19,20,50,51,55]. In particular, in [17] the authors have shown that m=Ω(klogn) number of measurements are sufficient to recover a signal from random phaseless observations, assuming that the signal lies on the range of a generative network with latent dimension *k*. The same authors have then provided in [23] a polynomial time algorithm for recovery under the previous settings. Note that, contrary to the sparse phase retrieval problem, generative priors for phase retrieval allow for efficient algorithms with optimal sample complexity, up to logarithmic factors, with respect to the intrinsic dimension of the signal.

Further theoretical advances in signal recovery with generative network priors have been spurred by using techniques from statistical physics. Recently, [30] analyzed the spiked matrix models (1) and (2) with $y_{🟉}$ in the range of a generative network with random weights, in the asymptotic limit k,n,N→∞ with n/k=O(1) and N/n=O(1). The analysis is carried out mainly for networks with sign or linear activation functions in the Bayesian setting where the latent vector is drawn from a separable distribution. The authors of [30] provide an Approximate Message Passing and a spectral algorithm, and they numerically observe no statistical-computational gap as these polynomial time methods are able to asymptotically match the information-theoretic optimum. In this asymptotic regime, [60] further provided precise statistical and algorithmic thresholds for compressed sensing and phase retrieval.

## 3. Algorithm and Main Result

In this section we present an efficient and statistically-optimal algorithm for the estimation of the signal $y_{🟉}$ given a spiked matrix *Y* of the form (1) or (2). The recovery method is detailed in Algorithm 1, and it is based on the direct optimization of the nonlinear least squares problem (4).

Applied in [16] for denoising and compressed sensing under generative network priors, and later used in [23] for phase retrieval, the first order optimization method described in Algorithm 1 leverages the theory of *Clarke subdifferentials* (the reader is referred to [61] for more details). As the objective function *f* is continuous and piecewise smooth, at every point $x\in R^{k}$ it has a Clarke subdifferential given by

(9) $$\partial f\left(x\right)=\mathrm{conv}\left\{v_{1},v_{2},\ldots ,v_{T}\right\},$$

where conv denotes the convex hull of the vectors $v_{1},\ldots ,v_{T}$, which are respectively the gradient of the *T* smooth functions adjoint at *x*. The vectors $v_{x}\in \partial f\left(x\right)$ are the *subgradients* of *f* at *x*, and at a point *x* where *f* is differentiable it holds that ∂f(x)={∇f(x)}.

**Figure 1:**
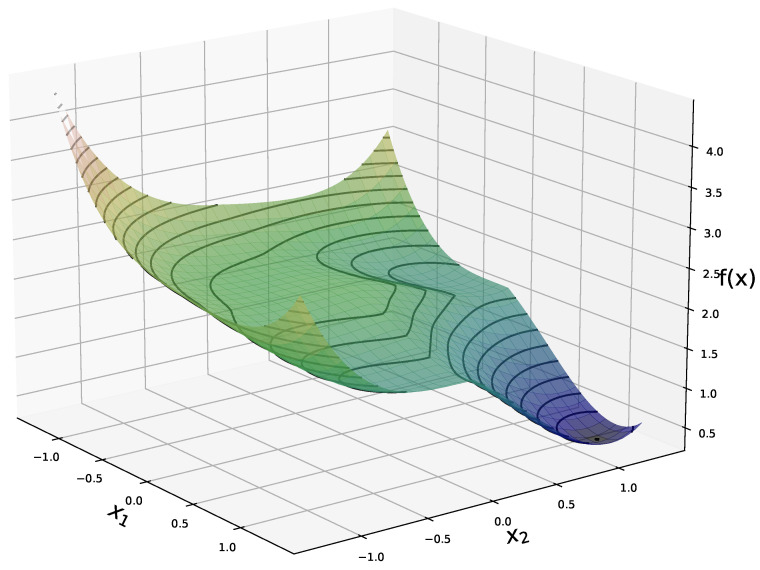
Expected value, with respect to the weights, of the objective function *f* in (4) in the noiseless case (see (16) for explicit formula), for a network with latent dimension k=2 and $x_{🟉}=\left[1,1\right]$.

The reconstruction method presented in Algorithm 1 is motivated by the landscape analysis of the minimization problem (4) for a network *G* with sufficiently-expansive Gaussian weights matrices. Under this assumption, we showed in [31] that (4) has a benign optimization geometry and in particular that for any nonzero point outside a neighborhood of $x_{🟉}$ and a negative multiple of it, any subgradient of *f* is a direction of strict descent. Furthermore we showed that the points in the vicinity of the spurious negative multiple of $x_{🟉}$ have function values strictly larger than those close to $x_{🟉}$. Figure 1 shows the expected value of *f* in the noiseless case, ν=0 and N→∞, for a generative network with latent dimension k=2. This plot highlights the global minimimum at $x_{🟉}=\left[1,1\right]$, and the flat region in near a negative multiple of $x_{🟉}$.

At each step, the subgradient method in Algorithm 1 checks if the current iterate $x_{i}$ has a larger loss value than its negative multiple, and if so negates $x_{i}$. As we show in the proof of our main result, this step will ensure that the algorithm will avoid the neighborhood around the spurious negative multiple of $x_{🟉}$ and will converge to the neighborhood around $x_{🟉}$ in a polynomial number of steps.

Below we make the following assumptions on the weight matrices of *G*.

**Assumption** **1.** *The generative network G defined in (3), has weights $W_{i}\in R^{n_{i}\times n_{i-1}}$ with i.i.d. entries from $N(0,1/n_{i})$ and satisfying the expansivity condition with constant ϵ>0:*

(10) $$n_{i+1}\geq c\;n_{i}\;\epsilon^{-2}\log\left(1/\epsilon \right)$$

*for all i and a universal constant c>0.*

We note that in [31] the expansivity condition was more stringent, requiring an additional log factor. Since the publication of our paper, [62] has shown that the more relaxed assumption (10) suffices for ensuring a benign optimization geometry. Under Assumption 1, our main theorem below shows that the subgradient method in Algorithm 1 can estimate the spike $y_{🟉}$ with optimal sample complexity and in a polynomial number of steps.

**Theorem** **1.** *Let $x_{🟉}\in R^{k}$ nonzero and $y_{🟉}=G\left(x_{🟉}\right)$ where G is a generative network satisfying Assumption 1 with $\epsilon \leq K_{1}/d^{96}$. Consider the minimization problem (4) and assume that the noise level ω satisfies $\omega \leq K_{2}{\left\|x_{🟉}\right\|}_{2}^{2}2^{-d}/d^{44}$ where:*

- *for the* ***Spiked Wishart Model*** *(1) take $M=\Sigma_{N}-\sigma^{2}I_{n}$, and*
  $$\omega :=\left(\|y_{🟉}{\|}_{2}^{2}+\sigma^{2}\right)\max\left\{\sqrt{\frac{338k\log(3\;n_{1}^{d}n_{2}^{d-1}\ldots n_{d-1}^{2}n)}{N}},\frac{156k\log(3\;n_{1}^{d}n_{2}^{d-1}\ldots n_{d-1}^{2}n)}{N}\right\};$$
- *for the* ***Spiked Wigner Model*** *(2) take M=Y, and*
  $$\omega :=\nu \sqrt{\frac{169k\log(3\;n_{1}^{d}n_{2}^{d-1}\ldots n_{d-1}^{2}n)}{n}}.$$

*Consider Algorithm 1 with nonzero $x_{0}$ and $\|x_{0}{\|}_{2}<R_{🟉}$ where $R_{🟉}\geq 5{\left\|x_{🟉}\right\|}_{2}/\left(2\sqrt{2}\right)$, and stepsize $\mu =2^{2d}K_{3}/\left(8d^{4}R_{🟉}^{2}\right)$. Then with probability at least $1-2e^{-k\log n}-\sum_{i=1}^{d}e^{-Cn_{i-1}}$, $0<\|x_{i}{\|}_{2}<R_{🟉}$ for any i≥1, there exists an integer $T\leq K_{4}f\left(x_{0}\right)2^{2d}/\left(R_{🟉}^{4}d^{4}\epsilon \right)$ such that for any i≥T:*

(11) $$\begin{array}{cc} \|x_{i+1}-x_{🟉}{\|}_{2} & \leq \rho_{1}^{i+1-T}{\left\|x_{T}-x_{🟉}\right\|}_{2}+\rho_{2}\;\frac{2^{d}}{\|x_{🟉}{\|}_{2}}\omega \end{array}$$

(12) $$\begin{array}{cc} \|G\left(x_{i+1}\right)-y_{🟉}{\|}_{2} & \leq \frac{1.2}{2^{d/2}}\rho_{1}^{i+1-T}{\left\|x_{T}-x_{🟉}\right\|}_{2}+1.3\rho_{2}\;\frac{\omega }{\|y_{🟉}{\|}_{2}} \end{array}$$

*where C>0, $K_{1},\ldots ,K_{4}>0$, $\rho_{1}\in \left(0,1\right)$ and $\rho_{2}>0$ are universal constants.*

Note that the quantity $2^{2d}$ in the hypotheses and conclusions of the theorem is an artifact of the scaling of the network and it should not be taken as requiring exponentially small noise or number of steps. Indeed under Assumption 1, the ReLU activation zeros out roughly half of the entries of its argument leading to an “effective” operator norm of approximately 1/2. We furthermore notice that the dependence of the depth *d* is likely quite conservative and it was not optimized in the proof as the main objective was to obtain tight dependence on the intrinsic dimension of the signal *k*. As shown in the numerical experiments, the actual dependence on the depth is much better in practice. Finally, observe that despite the nonconvex nature of the objective function in (4) we obtain a rate of convergence which is not directly dependent on the dimension of the signal, reminiscent of what happens in the convex case.

The quantity ω in Theorem 1 can be interpreted as the intrinsic noise level of the problem (inverse SNR). The theorem guarantees that in a polynomial number of steps the iterates of the subgradient method will converge to $x_{🟉}$ up to ω. For $\tilde{T}$ large enough $G(x_{\tilde{T}})$ will satisfy the rate-optimal error bounds (5) and (6).

### Numerical Experiments

We illustrate the predictions of our theory by providing results of Algorithm 1 on a set of synthetic experiments. We consider 2-layer generative networks with ReLU activation functions, hidden layer of dimension $n_{1}=500$, output dimension $n_{2}=n=1500$ and varying number of latent dimension k∈[40,60,100]. We randomly sample the weights of the matrix independently from $N(0,2/n_{i})$ (this scaling removes that $2^{d}$ dependence in Theorem 1). We then consider data *Y* according the spiked models (1) and (2), where $x_{🟉}\in R^{k}$ is chosen so that $y_{🟉}=G\left(x_{🟉}\right)$ has unit norm. For the Wishart model, we vary the number of samples *N*; and for the Wigner model, we vary the noise level ν so that the following quantities remain constant for the different networks with latent dimension *k*:

$$\theta_{\mathrm{WS}}:=\sqrt{k\log(n_{1}^{2}\;n)/N},\;\theta_{\mathrm{WG}}:=\nu \sqrt{k\log(n_{1}^{2}\;n)/n}.$$

**Figure 2:**
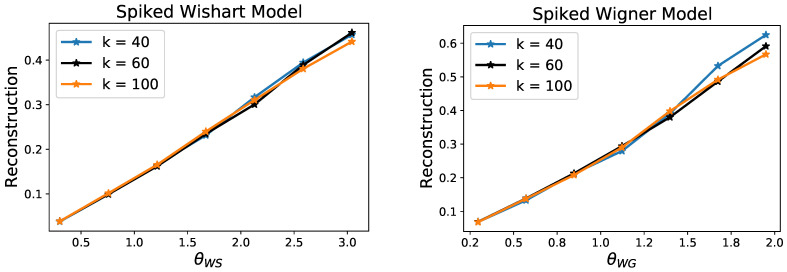
Reconstruction error for the recovery of a spike $y_{🟉}=G\left(x_{🟉}\right)$ in the Wishart and Wigner models with random generative network priors. Each point corresponds to the average over 50 random drawing of the network weights and samples. These plots demonstrate that the reconstruction errors follow the scalings established by Theorem 1.

In Figure 2 we plot the reconstruction error given by $\|G\left(x\right)-y_{🟉}{\|}_{2}$ against $\theta_{\mathrm{WS}}$ and $\theta_{\mathrm{WG}}$. As predicted by Theorem 1, the errors scale linearly with respect to these control parameters, and moreover the overlap of these plots confirms that these rates are tight with respect to the order of *k*.

## 4. Recovery Under Deterministic Conditions

We will derive Theorem 1 from Theorem 3, below, which is based on a set of deterministic conditions on the weights of the matrix and the noise. Specifically, we consider the minimization problem (4) with

$$M=G\left(x_{🟉}\right)G{\left(x_{🟉}\right)}^{T}+H$$

for an unknown symmetric matrix $H\in R^{n\times n}$, nonzero $x_{🟉}\in R^{k}$, and a given *d*-layer feed forward generative network *G* as in (3).

In order to describe the main deterministic conditions on the generative network *G*, we begin by introducing some notation. For $W\in R^{n\times k}$ and $x\in R^{k}$, we define the operator $W_{+,x}:=\mathrm{diag}\left(Wx>0\right)W$ such that $\mathrm{relu}\left(Wx\right)=W_{+,x}x$. Moreover, we let $W_{1,+,x}={\left(W_{1}\right)}_{+,x}=\mathrm{diag}\left(W_{1}x>0\right)W_{1}$, and for 2≤i≤d we define recursively

$$W_{i,+,x}=\mathrm{diag}\left(W_{i},\Pi_{j=i-1}^{1}W_{j,+,x}x>0\right)W_{i},$$

where $\Pi_{i=d}^{1}W_{i}=W_{d}W_{d-1}\ldots W_{1}$. Finally we let $\Lambda_{x}=\;\Pi_{j=d}^{1}W_{j,+,x}$ and note that $G\left(x\right)=\Lambda_{x}x$. With this notation we next recall the following deterministic condition on the layers of the generative network.

**Definition** **2** (Weight Distribution Condition [15]). *We say that $W\in R^{n\times k}$ satisfies the Weight Distribution Condition (WDC) with constant ϵ>0 if for all nonzero $x_{1},x_{2}\in R^{k}$:*

$$\|W_{+,x_{1}}^{T}W_{+,x_{2}}-Q_{x_{1},x_{2}}{\|}_{2}\leq \epsilon ,$$

*where*

$$Q_{x_{1},x_{2}}=\frac{\pi -\theta_{x_{1},x_{2}}}{2\pi }I_{k}+\frac{\sin\theta_{x_{1},x_{2}}}{2\pi }M_{{\hat{x}}_{2}↔{\hat{x}}_{2}}$$

*and $\theta_{x_{1},x_{2}}=∠\left(x_{1},x_{2}\right)$, ${\hat{x}}_{1}=x_{1}/{\left\|x_{1}\right\|}_{2}$, ${\hat{x}}_{2}=x_{2}/{\left\|x_{2}\right\|}_{2}$, $I_{k}$ is the k×k identity matrix and $M_{{\hat{x}}_{1}↔{\hat{x}}_{2}}$ is the matrix that sends ${\hat{x}}_{1}\mapsto {\hat{x}}_{2}$, ${\hat{x}}_{2}\mapsto {\hat{x}}_{1}$, and with kernel span ${\left(\left\{x_{1},x_{2}\right\}\right)}^{⊥}$.*

Note that $Q_{x_{1},x_{2}}$ is the expected value of $W_{+,x_{1}}^{T}W_{+,x_{2}}$ when *W* has rows $w_{i}∼N\left(0,I_{k}/n\right)$, and if $x_{1}=x_{2}$ then $Q_{x_{1},y_{2}}$ is an isometry up to the scaling factor 1/2. Below we will say that a *d*-layer generative network *G* of the form (3), satisfies the WDC with constant ϵ>0 if every weight matrix $W_{i}$ has the WDC with constant ϵ for all i=1,…d.

The WDC was originally introduced in [15], and ensures that the angle between two vectors in the latent space is approximately preserved at the output layer and, in turn, it guarantees the invertibility of the network. Assumption 1 will guarantees that the generative network *G* satisfies the WDC with high probability.

We are now able to state our recovery guarantees for a spike $y_{🟉}$ under deterministic conditions on the network *G* and noise *H*.

**Theorem** **3.** *Let d≥2 and assume the generative network (3) has weights $W_{i}\in R^{n_{i}\times n_{i-1}}$ satisfying the WDC with constant $0<\epsilon \leq K_{1}/d^{96}$. Consider Algorithm 1 with $M=G\left(x_{🟉}\right)G{\left(x_{🟉}\right)}^{T}+H$, $x_{🟉}\in R^{k}\backslash \left\{0\right\}$ and H a symmetric matrix satisfying:*

(13) $$∥\Lambda_{x}^{T}H\Lambda_{x}{∥}_{2}\leq \frac{\omega }{2^{d}},\;\mathrm{and}\;\omega \leq K_{2}\frac{∥x_{🟉}{∥}_{2}^{2}2^{-d}}{d^{44}}.$$

*Take $x_{0}$ nonzero and with $\|x_{0}{\|}_{2}<R_{🟉}$ where $R_{🟉}\geq 5{\left\|x_{🟉}\right\|}_{2}/\left(2\sqrt{2}\right)$, $\mu =2^{2d}K_{3}/\left(8d^{4}R_{🟉}^{2}\right)$. Then the iterates ${\left\{x_{i}\right\}}_{i\geq 0}$ generated by the Algorithm 1 satisfy $0<\|x_{i}\|<R_{🟉}$ and obey to the following:*

*(A)*  *there exists an integer $T\leq K_{4}\frac{f\left(x_{0}\right)2^{2d}}{R_{🟉}^{4}d^{4}\epsilon }$ such that*
  $$∥x_{T}-x_{🟉}{∥}_{2}\leq K_{5}d^{14}\sqrt{\epsilon }∥x_{🟉}{∥}_{2}+K_{6}2^{d}d^{10}\omega {∥x_{🟉}∥}_{2}^{-1}$$
*(B)*  *for any i≥T:*
  (14) $$\begin{array}{cc} ∥x_{i+1}-x_{🟉}{∥}_{2} & \leq \rho_{1}^{i+1-T}{∥x_{T}-x_{🟉}∥}_{2}+\rho_{2}\;\frac{2^{d}}{∥x_{🟉}{∥}_{2}}\omega \end{array}$$
  (15) $$\begin{array}{cc} \|G\left(x_{i+1}\right)-G\left(x_{🟉}\right){\|}_{2} & \leq \frac{1.2}{2^{d/2}}\rho_{1}^{i+1-T}{\left\|x_{T}-x_{🟉}\right\|}_{2}+1.3\rho_{2}\;\frac{\omega }{\|y_{🟉}{\|}_{2}} \end{array}$$
  *where $K_{1},\ldots ,K_{6}>0$, $\rho_{1}\in \left(0,1\right)$ and $\rho_{2}>0$ are universal constants.*

Theorem 1 follows then from Theorem 3 after proving that with high probability the spectral norm of $\Lambda_{x}^{T}H\Lambda_{x}$, where $H=M-y_{🟉}{y_{🟉}}^{T}$, can be upper bounded by $\omega /2^{d}$, and the weights of the network *G* satisfy with high probability the WDC.

In the rest of this section section we will describe the main steps and tools needed to prove Theorem 3.

### 4.1. Technical Tools and Outline of the Proofs

Our proof strategy for Theorem 3 can be summarized as follows:

1. In Proposition A1 (Appendix A.3) we show that the iterates ${\left\{x_{i}\right\}}_{i=1}$ of the Algorithm 1 stay inside the Euclidean ball of radius $R_{🟉}$ and remain nonzero for all i≥1.
2. We then identify two small Euclidean balls $B_{+}$ and $B_{-}$ around respectively $x_{🟉}$ and $-\rho_{d}x_{🟉}$, where $\rho_{d}\in \left(0,1\right)$ only depends on the depth of the network. In Proposition A2 we show that after a polynomial number of steps, the iterates $\left\{x_{i}\right\}$ of the Algorithm 1 enter the region $B_{+}\cup B_{-}$ (Appendix A.4).
3. We show, in Proposition A3, that the negation step causes the iterates of the algorithm to avoid the spurious point $-\rho_{d}x_{🟉}$ and actually enter $B_{+}$ within a polynomial number of steps (Appendix A.5).
4. We finally show in Proposition A4, that in $B_{+}$ the loss function *f* enjoys a favorable convexity-like property, which implies that the iterates $\left\{x_{i}\right\}$ will remain in $B_{+}$ and eventually converge to $x_{🟉}$ up to the noise level (Appendix A.6).

One of the main difficulties in the analysis of a subgradient method in Algorithm 1 is the lack of smoothness of the loss function *f*. We show that the WDC allows us to overcome this issue by showing that the subgradients of *f* are uniformly close, up to the noise level, to the vector field $h_{x}\in R^{k}$:

$$h_{x}:=\frac{1}{2^{2d}}\left(xx^{T}-{\tilde{h}}_{x}{\tilde{h}}_{x}^{T}\right)x,$$

where ${\tilde{h}}_{x}$ is continuous for nonzero *x* (see Appendix A.2). We show furthermore that $h_{x}$ is locally Lipschitz, which allows us to conclude that the gradient method decreases the value of the loss function until eventually reaching $B_{+}\cup B_{-}$ (Appendix A.4).

Using the WDC, we show that the loss function *f* is uniformly close to

(16) $$f_{E}\left(x\right)=\frac{1}{2^{2d+2}}\left({\left\|x\right\|}_{2}^{4}+{\left\|x_{🟉}\right\|}_{2}^{4}-2{\langle x,{\tilde{h}}_{x}\rangle }^{2}\right).$$

A direct analysis of $f_{E}$ reveals that its values inside $B_{-}$ are strictly larger then those inside $B_{+}$. This property extends to *f* as well, and guarantees that the gradient method will not converge to the spurious point $-\rho_{d}x_{🟉}$ (Appendix A.5).

## Appendix A. Supporting Lemmas and Proof of Theorem 3

The proof of Theorem 3 is provided in Appendix A.7. We begin this section with a set of preliminary results and supporting lemmas.

### Appendix A.1. Notation

We collect the notation that is used throughout the paper. For any real number *a*, let relu(a)=max(a,0) and for any vector $v\in R^{n}$, denote the entrywise application of relu as relu(v). Let diag(Wx>0) be the diagonal matrix with *i*-th diagonal element equal to 1 if ${\left(Wx\right)}_{i}>0$ and 0 otherwise. For any vector *x* we denote with ‖x‖ its Euclidean norm and for any matrix *A* we denote with ‖A‖ its spectral norm and with ${\left\|A\right\|}_{F}$ its Frobenius norm. The euclidean inner product between two vectors *a* and *b* is 〈a,b〉, while for two matrices *A* and *B* their Frobenius inner product will be denoted by ${\langle A,B\rangle }_{F}$. For any nonzero vector $x\in R^{n}$, let $\hat{x}=x/\left\|x\right\|$. For a set *S* we will write |S| for its cardinality and $S^{c}$ for its complement. Let B(x,r) be the Euclidean ball of radius *r* centered at *x*, and $S^{k-1}$ be the unit sphere in $R^{k}$. Let $\theta_{0}=∠\left(x,x_{🟉}\right)$ and for i≥0 let $\theta_{i+1}=g\left(\theta_{i}\right)$ where *g* is defined in (A1). We will write γ=Ω(δ) to mean that there exists a positive constant *C* such that γ≥Cδ and similarly γ=O(δ) if γ≤Cδ. Additionally we will use $a=b+O_{1}\left(\delta \right)$ when ‖a−b‖≤δ, where the norm is understood to be the absolute value for scalars, the Euclidean norm for vectors and the spectral norm for matrices.

### Appendix A.2. Preliminaries

For later convenience we will define the following vectors:

$$\begin{array}{cc} p_{x} & :=\Lambda_{x}^{T}\Lambda_{x}x,\\ q_{x} & :=\Lambda_{x}^{T}\Lambda_{x_{🟉}}x_{🟉},\\ {\bar{v}}_{x} & :=[p_{x}p_{x}^{T}-q_{x}q_{x}^{T}]x,\\ \eta_{x} & :=\Lambda_{x}^{T}H\Lambda_{x}x. \end{array}$$

Note then that when *f* is differentiable at *x*, then ${\tilde{v}}_{x}:=\nabla f\left(x\right)={\bar{v}}_{x}-\eta_{x}$ and in particular when H=0 then we have ${\tilde{v}}_{x}={\bar{v}}_{x}$.

The following function controls how the angles are contracted by a ReLU layer:

(A1) $$g\left(\theta \right):=\cos^{-1}\left(\frac{(\pi -\theta )\cos\theta +\sin\theta }{\pi }\right).$$

As we mentioned in Section 4.1, our analysis is based on showing that the subgradients of *f* are uniformly close to the vector field $h_{x}$ given by

(A2) $$h_{x}:=\frac{1}{2^{2d}}\left(xx^{T}-{\tilde{h}}_{x}{\tilde{h}}_{x}^{T}\right)x,$$

where

(A3) $${\tilde{h}}_{x}:=(\prod_{i=0}^{d-1}\frac{\pi -{\bar{\theta }}_{i}}{\pi })x_{🟉}+\sum_{i=1}^{d-1}\frac{\sin\theta_{i}}{\pi }(\prod_{j=i+1}^{d-1}\frac{\pi -{\bar{\theta }}_{j}}{\pi })\frac{\|x_{🟉}\|}{\left\|x\right\|}x,$$

and $\theta_{i}:=g\left({\bar{\theta }}_{i-1}\right)$ for *g* given by (A1) and $\theta_{0}=∠\left(x,y\right)$.

**Lemma** **A1** (Lemma 8 in [15]). *Suppose that d≥2 and the WDC holds with $\epsilon <1/{\left(16\pi d^{2}\right)}^{2}$, then for all nonzero $x,x_{🟉}\in R^{k}$,*

(A4) $$\begin{array}{cc} \langle \Lambda_{x}x,\Lambda_{x_{🟉}}x_{🟉}\rangle & \geq \frac{1}{4\pi }\frac{1}{2^{d}}{\left\|x\right\|}_{2}\left\|x_{🟉}\right\|, \end{array}$$

(A5) $$\begin{array}{cc} ∥\Lambda_{x}^{T}\Lambda_{x_{🟉}}x_{🟉}-\frac{{\tilde{h}}_{x}}{2^{d}}∥ & \leq 24\frac{d^{3}\sqrt{\epsilon }}{2^{d}}∥x_{🟉}∥,\;\mathrm{and} \end{array}$$

(A6) $$\begin{array}{cc} ∥\Lambda_{x}{∥}^{2}\leq \frac{1}{2^{d}}{\left(1+2\epsilon \right)}^{d}\leq \frac{1+4\epsilon d}{2^{d}} & \leq \frac{13}{12}\frac{1}{2^{d}}. \end{array}$$

**Proof.** The first two bounds can be found in [15] (Lemma 8). The third bound follows by noticing that the WDC implies:

$$∥\Lambda_{x}{∥}^{2}\leq \Pi_{i=d}^{1}{\left\|W_{i,+,x}\right\|}^{2}\leq \frac{1}{2^{d}}{\left(1+2\epsilon \right)}^{d}\leq \frac{1+4\epsilon d}{2^{d}}\leq \frac{13}{12}\frac{1}{2^{d}},$$

where we used log(1+z)≤z and $e^{z}\leq \left(1+2z\right)$ for all 0≤z≤1. □

The next lemma shows that the noiseless gradient ${\bar{v}}_{x}$ concentrates around $h_{x}$.

**Lemma** **A2.**
*Suppose d≥2 and the WDC holds with $\epsilon <1/{\left(16\pi d^{2}\right)}^{2}$, then for all nonzero $x,x_{🟉}\in R^{k}$:*

$$∥{\bar{v}}_{x}-h_{x}∥\leq 86\frac{d^{4}\sqrt{\epsilon }}{2^{2d}}\max(∥x_{🟉}{∥}^{2}{,∥x∥}^{2})∥x∥.$$

We now use the characterization of the Clarke subdifferential given in (9) to derive a bound on the concentration of $v_{x}\in \partial f\left(x\right)$ around $h_{x}$ up to the noise level.

**Lemma** **A3.**
*Under the assumptions of Lemma A2, and assuming $∥\Lambda_{x}^{T}H\Lambda_{x}\|\leq \omega /2^{d}$, for any $v_{x}\in \partial f\left(x\right)$:*

$$∥v_{x}-h_{x}\|\leq 86\frac{d^{4}\sqrt{\epsilon }}{2^{2d}}\max(\|x_{🟉}{\|}^{2}{,\|x\|}^{2})\|x\|+\frac{\omega }{2^{d}}\;\left\|x\right\|$$

From the above and the bound on the noise level ω we can bound the norm of the step $v_{x}$ in the Algorithm 1.

**Lemma** **A4.**
*Under the assumptions of Lemma A3, and assuming that ω satisfies (13), for any $v_{x}\in \partial f\left(x\right)$:*

$$∥v_{x}\|\leq \frac{4}{2^{2d}}d^{2}{\max(\|x\|}^{2},\|x_{🟉}{\|}^{2})\|x\|.$$

### Appendix A.3. Iterates Stay Bounded

In this section we prove that all the iterates $\left\{x_{i}\right\}$ generated by Algorithm 1 remain inside the Euclidean ball $B(0,R_{🟉})$ where $R_{🟉}\geq \sqrt{2}C_{🟉}\left\|x_{🟉}\right\|$ and $C_{🟉}=5/4$.

**Lemma** **A5.**
*Let the assumptions of Lemma A4 be satisfied, $\mu =K_{3}2^{2d}/\left(8d^{4}R_{🟉}^{2}\right)$ with $512\pi^{2}K_{3}<1$. Then for any $x\in R^{k}$ with $C_{🟉}\|x_{🟉}\left\|<\|x\right\|\leq R_{🟉}$ and any λ∈[0,1], it holds that $\|x-\lambda \;\mu \;v_{x}\left\|\leq \|x\right\|$.*

From the previous lemma we can now derive the boundedness of the iterates of the Algorithm 1.

**Proposition** **A1.**
*Under the assumptions of Theorem 3, if $x\in B\left(0,R_{🟉}\right)\backslash \left\{0\right\}$ it follows that $x-\lambda \;\mu \;v_{x}\in B\left(0,R_{🟉}\right)\backslash \left\{0\right\}$. Furthermore if $x_{0}\in B\left(0,R_{🟉}\right)\backslash \left\{0\right\}$, the iterates ${\left\{x_{i}\right\}}_{i=1}$ of the Algorithm 1 satisfy $x_{i}-\lambda \;\mu \;v_{x_{i}}\in B\left(0,R_{🟉}\right)\backslash \left\{0\right\}$ for all i≥1 and λ∈[0,1].*

**Proof.** Assume $C_{🟉}\|x_{🟉}\left\|<\|x\right\|\leq R_{🟉}$, then the conclusions follows from Lemma A5. Assuming instead that $\left\|x\right\|\leq C_{🟉}\left\|x_{🟉}\right\|$, note that

$$\|x-\lambda \;\mu \;v_{x}\left\|\leq \|x\|+\mu \right\|v_{x}\|\leq \left(1+\frac{1}{2d^{2}}\right)\left\|x\right\|\leq R_{🟉}$$

using Lemma A4, d≥2 and the assumptions on μ and $R_{🟉}$. Finally observe that if $x_{i}\in B\left(0,R_{🟉}\right)$ then the same holds for ${\tilde{x}}_{i}$. Finally if for some i≥0 it was the case that $0={\tilde{x}}_{i}-\lambda \mu v_{{\tilde{x}}_{i}}$, then this would imply that $\|{\tilde{x}}_{i}\left\|=\lambda \mu \right\|v_{{\tilde{x}}_{i}}\|$ which cannot happen because by Lemma A4 and the choice of the step size it holds that $\mu \|v_{{\tilde{x}}_{i}}\left\|\leq \right\|{\tilde{x}}_{i}\|/8$. □

### Appendix A.4. Convergence to $S_{\beta }$

We define the set $S_{\beta }$ outside which we can lower bound the norm of $\|h_{x}\|$ as

$$S_{\beta }:=\{x\in R^{k}\left|\;\right\|h_{x}\|\leq \frac{\beta }{2^{2d}}{\max(\|x\|}^{2},\|{x_{🟉}\|}^{2})\|x\|\},$$

where we take

(A7) $$\beta =7\cdot \left(86d^{4}\sqrt{\epsilon }+2^{d}\omega {\left\|x_{🟉}\right\|}^{-2}\right).$$

Outside the set $S_{\beta }$ the sub-gradients of *f* are bounded below and the landscape has favorable optimization geometry.

**Lemma** **A6.**
*Let $x\in B\left(0,R_{🟉}\right)\backslash \left\{0\right\}\cap S_{\beta }^{c}$, then for all $v_{x}\in \partial f\left(x\right)$*

(A8) $$∥v_{x}\|\geq 6\left(\frac{\max(\|x{\|}^{2},\|x_{🟉}{\|}^{2})\|x\|}{2^{2d}}86d^{4}\sqrt{\epsilon }+\frac{\omega }{2^{d}}\left\|x\right\|\right).$$

*Moreover let λ∈[0,1] and $x_{\lambda }=x-\lambda \mu v_{x_{\lambda }}$ then*

(A9) $$∥v_{x}-v_{x_{\lambda }}\|\leq \frac{15}{16}\left\|v_{x}\right\|,$$

*for all $v_{x}\in \partial f\left(x\right)$ and $v_{x_{\lambda }}\in \partial f\left(x_{\lambda }\right)$.*

Based on the previous lemma we can prove the main result of this section.

**Proposition** **A2.**
*Under the assumptions of Theorem 3, if $x_{i}\in B\left(0,R_{🟉}\right)\backslash \left\{0\right\}\cap S_{\beta }^{c}$ then*

(A10) $$f\left(x_{i+1}\right)-f\left(x_{i}\right)\leq -\frac{KR_{🟉}^{4}d^{4}\epsilon }{2^{2d}}$$

*for some numerical constant K>0. Moreover there exists an integer $T\leq \frac{f\left(x_{0}\right)2^{2d}}{KR_{🟉}^{4}d^{4}\epsilon }$ such $x_{i+T}\in S_{\beta }$.*

**Proof.** Let $x_{i}\notin S_{\beta }$ and assume that $f\left(x_{i}\right)\leq f\left(-x_{i}\right)$, then ${\tilde{x}}_{i}=x_{i}$. By the mean value theorem for Clarke subdifferentials [61] (Theorem 8.13), there exists λ∈(0,1) such that for $x_{i,\lambda }=x_{i}-\mu \lambda v_{x_{i}}$ and a $v_{x_{i,\lambda }}\in \partial f\left(x_{i,\lambda }\right)$ it holds that

(A11) $$\begin{array}{cc} f\left(x_{i+1}\right)-f\left({\tilde{x}}_{i}\right) & =\langle v_{x_{i,\lambda }},-\mu v_{x_{i}}\rangle \\ \; & =\langle v_{x_{i}},-\mu v_{x_{i}}\rangle +\langle v_{x_{i,\lambda }}-v_{x_{i}},-\mu v_{x_{i}}\rangle \\ \; & \leq -\mu \|v_{x_{i}}\left\|(\right\|v_{x_{i}}\left\|-\right\|v_{x_{i,\lambda }}-v_{x_{i}}\left\|\right)\\ \; & \leq -\frac{\mu }{16}{\left\|v_{x_{i}}\right\|}^{2} \end{array}$$

where the first inequality follows from the triangle inequality and the second from Equation (A9). Next observe that by (A8)

$$∥v_{x}{∥}^{2}\geq 36\frac{{\max(\|x\|}^{2},\|x_{🟉}{\|}^{2}{)}^{2}{\left\|x\right\|}^{2}}{2^{4d}}86^{2}d^{8}\epsilon$$

which together with the definition of μ and (A11) gives (A10). Next take $x_{i}\notin S_{\beta }$ and assume $f\left(x_{i}\right)>f\left(-x_{i}\right)$, so that ${\tilde{x}}_{i}=-x_{i}$. Observe that

$$f\left(x_{i+1}\right)-f\left(x_{i}\right)<f\left(x_{i+1}\right)-f\left(-x_{i}\right)=f\left(x_{i+1}\right)-f\left({\tilde{x}}_{i}\right),$$

we obtain then (A10) proceeding as before. Finally the claim on the maximum number of iterations directly follows by a telescopic sum on (A10) and f(·)≥0. □

### Appendix A.5. Convergence to a Neighborhood Around $x_{🟉}$

In the previous section we have shown that after a finite number of steps the iterates $\left\{x_{i}\right\}$ of the Algorithm 1 will enter in the region $S_{\beta }$. In this section we show that, thanks to the negation step in the descent algorithm, they will eventually be confined in a neighborhood of $x_{🟉}$.

The following lemma shows that $S_{\beta }$ is contained inside two balls around $x_{🟉}$ and $-x_{🟉}$.

**Lemma** **A7.**
*Suppose $8\pi d^{6}\sqrt{\beta }\leq 1$, then we have $S_{\beta }\subset B^{+}\cup B^{-}$ where*

$$B^{+}:=B(x_{🟉},R_{1}\beta d^{10}\|x_{🟉}\|)\;\mathrm{and}\;B^{-}:=B(-\rho_{d}x_{🟉},R_{2}\sqrt{\beta }d^{10}\|x_{🟉}\left\|\right),$$

*where $R_{1},R_{2}$ are numerical constants and $0<\rho_{d}<1$ such that $\rho_{d}\to 1$ as d→∞.*

We furthermore observe that the ball around $-x_{🟉}$ has values strictly higher that the one around $x_{🟉}$.

**Lemma** **A8.**
*Suppose that d≥2, the WDC holds with $\epsilon <1/{\left(16\pi d^{2}\right)}^{2}$ and H satisfies (13). Then for any $\phi_{d}\in \left[\rho_{d},1\right]$, it holds that*

(A12) f(x)<f(y),

*for all $x\in B(\phi_{d}x_{🟉},\varrho \|x_{🟉}\|d^{-12})$ and $y\in B(-\phi_{d}x_{🟉},\varrho \|x_{🟉}\|d^{-12})$ where ϱ<1 is a universal constant.*

The main result of this section is about the convergence to a neighborhood of $x_{🟉}$ of the iterates $\left\{x_{i}\right\}$.

**Proposition** **A3.**
*Under the assumptions of Theorem 3, if $x_{i}\in B\left(0,R_{🟉}\right)\backslash \left\{0\right\}$, then there exists a finite number of steps $T\leq K\frac{f\left(x_{i}\right)2^{2d}}{R_{🟉}^{4}d^{4}\epsilon }$ such that $x_{i+T}\in B(x_{🟉},R_{1}\beta d^{10}\|x_{🟉}\left\|\right)$. In particular it holds that*

(A13) $$∥x_{i+T}-x_{🟉}\|\leq C_{1}d^{14}\sqrt{\epsilon }\|x_{🟉}\|+C_{2}2^{d}d^{10}\omega {\left\|x_{🟉}\right\|}^{-1}.$$

**Proof.** Either $x_{i}\in S_{\beta }$ or by Proposition A2 there exist $T^{\prime }$ such that $x_{i+T^{\prime }}\in S_{\beta }$. By the choice of ϵ>0, the definition of β in (A7), and the assumption on the noise level (13), it follows that the hypotheses of Lemma A7 are satisfied. We therefore define the two neighborhoods $S_{\beta }^{+}:=S_{\beta }\cap B^{+}$ and $S_{\beta }^{-}:=S_{\beta }\cap B^{-}$ and conclude that that either $x_{i+T^{\prime }}\in S_{\beta }^{+}$ or $x_{i+T^{\prime }}\in S_{\beta }^{-}$. We next notice that $S_{\beta }^{+}\subseteq B(x_{🟉},\varrho \|x_{🟉}\left\|d^{-12}\right)\;$ and $\;S_{\beta }^{-}\subseteq B(-\rho_{d}x_{🟉},\varrho \|x_{🟉}\left\|d^{-12}\right)$. We can then use Lemma A8 to conclude that by the negation step, if $x_{i+T^{\prime }}\in S_{\beta }^{+}$ then ${\tilde{x}}_{i+T^{\prime }}\in S_{\beta }^{+}$, otherwise we will have ${\tilde{x}}_{i+T^{\prime }}\in -S_{\beta }^{-}$. We now analyze the case $x_{i+T^{\prime }}\in -S_{\beta }^{-}\cap S_{\beta }^{c}$. Applying again Proposition A2, we have that there exists and integer $T^{\prime \prime }\leq K\frac{f\left(x_{i+N}\right)2^{2d}}{R_{🟉}^{4}d^{4}\epsilon }$ such that $x_{i+T^{\prime }+T^{\prime \prime }}\in S_{\beta }$. Furthermore Proposition A2 implies that $f\left(x_{i+T^{\prime }+T^{\prime \prime }}\right)<f\left(x_{i+T^{\prime }}\right)$, while from Lemma A8 we know that $f\left(x_{i+T^{\prime }}\right)<f\left(y\right)$ for all $y\in S_{\beta }^{-}$. We conclude therefore that $x_{i+T^{\prime }+T^{\prime \prime }}$ must be in $S_{\beta }^{+}$. In summary we obtained that there exists an integer $T\leq K\frac{f\left(x_{0}\right)2^{2d}}{R_{🟉}^{4}d^{4}\epsilon }$ such that $x_{i+T}\in S_{\beta }^{+}\subset B^{+}$. Finally Equation (A13) follows from the definition of β in (A7) and $B^{+}$. □

### Appendix A.6. Convergence to $x_{🟉}$ up to Noise

**Lemma** **A9.**
*Suppose the WDC holds with $\epsilon <1/(200^{4}d^{6})$, then for all $x\in B(x_{🟉},d\sqrt{\epsilon }\|x_{🟉}\|)$ and $v_{x}\in \partial_{x}f\left(x\right)$, it holds that*

$$∥v_{x}-\frac{\tau_{1}}{2^{2d}}{\left\|x_{🟉}\right\|}^{2}\left(x-x_{🟉}\right)\|\leq \tau_{2}\frac{\|x_{🟉}{\|}^{2}}{2^{2d}}\|x-x_{🟉}\|+\tau_{3}\frac{\omega }{2^{d}}\left\|x_{🟉}\right\|$$

*where $\tau_{1}=21/5$, $\tau_{2}=17/5$ and $\tau_{3}=\left(1+1/{\left(400\right)}^{2}\right)$.*

Based on the previous condition on the direction of the sub-gradients we can then prove that the iterates of the algorithm converge to $x_{🟉}$ up to noise.

**Proposition** **A4.**
*Under the assumptions of Theorem (3), if $x_{T}\in B^{+}$ then for any i≥T it holds that $x_{i}\in B^{+}$ and furthermore*

(A14) $$\begin{array}{cc} \|x_{i+1}-x_{🟉}\| & \leq \rho_{1}^{i+1-T}\left\|x_{T}-x_{🟉}\right\|+\rho_{2}\;\frac{2^{d}}{\|x_{🟉}\|}\omega \end{array}$$

*where $\rho_{1}\in \left(0,1\right)$ and $\rho_{2}>0$ are numerical constants.*

**Proof.** If i=T, by Proposition A2, it follows that $x_{i}={\tilde{x}}_{i}\in B^{+}$. Furthermore, the assumptions of Lemma A9 are satisfied and we can write:

(A15) $$\begin{array}{cc} \|x_{i+1}-x_{🟉}\| & =\|{\tilde{x}}_{i}-\mu v_{{\tilde{x}}_{i}}-x_{🟉}\|\\ \; & \leq (1-\frac{\mu \tau_{1}{\left\|x_{🟉}\right\|}^{2}}{2^{2d}})\|{\tilde{x}}_{i}-x_{🟉}\left\|+\mu \right\|v_{{\tilde{x}}_{i}}-\frac{\tau_{1}}{2^{2d}}\|x_{🟉}{\|}^{2}\left({\tilde{x}}_{i}-x_{🟉}\right)\|\\ \; & \leq (1-\frac{\mu \left(\tau_{1}-\tau_{2}\right){\left\|x_{🟉}\right\|}^{2}}{2^{2d}})\|{\tilde{x}}_{i}-x_{🟉}\|+\mu \;\tau_{3}\;\frac{\omega }{2^{d}}\left\|x_{🟉}\right\|. \end{array}$$

Next recall that ω satisfies (13), $\mu =K_{3}2^{2d}/\left(8d^{4}R_{🟉}^{2}\right)$ and $R_{🟉}^{2}\geq 25{\left\|x_{🟉}\right\|}^{2}/8$, then

$$\begin{array}{cc} \|x_{i+1}-x_{🟉}\| & \leq (1-\frac{\mu \left(\tau_{1}-\tau_{2}\right){\left\|x_{🟉}\right\|}^{2}}{2^{2d}})\|{\tilde{x}}_{i}-x_{🟉}\|+\mu \;\frac{K_{2}}{2^{2d}}\frac{\tau_{3}}{d^{44}}{\left\|x_{🟉}\right\|}^{3}\\ & \leq [1-\left(\tau_{1}-\tau_{2}\right)\frac{K_{3}}{25d^{4}}+K_{2}\frac{K_{3}}{25d^{48}}]\left\|x_{🟉}\right\| \end{array}$$

Therefore for $K_{2}$ and $K_{3}$ small enough we obtain that $x_{i+1}\in B(x_{🟉},R_{1}\;\beta \;d^{10}\|x_{🟉}\left\|\right)$ and by induction this holds for all i≥T. Finally we obtain (A14) by letting $\rho_{1}=\left(1-\mu \left(\tau_{1}-\tau_{2}\right){\left\|x_{🟉}\right\|}^{2}/2^{2d}\right)$ and $\rho_{2}=K_{3}\tau_{3}/\left(25d^{4}\right)$ in (A15). □

### Appendix A.7. Proof of Theorem 3

We begin recalling the following fact on the local Lipschitz property of the generative network *G* under the WDC.

**Lemma** **A10** (Lemma 21 in [16]). *Suppose $x\in B(x_{🟉},d\sqrt{\epsilon }\|x_{🟉}\|)$, and the WDC holds with $\epsilon <1/(200^{4}d^{6})$. Then it holds that:*

$$\|G\left(x\right)-G\left(x_{🟉}\right)\|\leq \frac{1.2}{2^{d/2}}\left\|x-x_{🟉}\right\|.$$

We then conclude the proof of Theorem 3 by using the above lemma and the results in the previous sections.

(I) By assumption $x_{0}\in B\left(0,R_{🟉}\right)\backslash \left\{0\right\}$ so that according to Proposition A1 for any i≥1 it holds that $x_{i}\in B\left(0,R_{🟉}\right)\backslash \left\{0\right\}$.
(II) By Proposition A3, there exists an integer *T* such that $x_{T}\in B^{+}$ and therefore it satisfies the conclusions of Theorem 3.A
  $$\|x_{T}-x_{🟉}\|\leq C_{1}d^{14}\sqrt{\epsilon }\|x_{🟉}\|+C_{2}2^{d}d^{10}\omega {\left\|x_{🟉}\right\|}^{-1}.$$
(III) Once in $B^{+}$ the iterates of Algorithm 1 converge to $x_{🟉}$ up to the noise level, as shown by Proposition A4 and Equation (A14)
  $$∥x_{i+1}-x_{🟉}\|\leq \rho_{1}^{i+1-T}\left\|x_{T}-x_{🟉}\right\|+\rho_{2}\;\frac{2^{d}}{\|x_{🟉}\|}\omega$$
  which corresponds to (11) in Theorem 3.B.
(IV) The reconstruction error (12) in Theorem 3.B, follows then from (11) by applying Lemma A10 and the lower bound (A4).

## Appendix B. Supplementary Proofs

### Appendix B.1. Supplementary Proofs for Appendix A.2

Below we prove Lemma A2 on the concentration of the gradient of *f* at a differentiable point.

**Proof of Lemma A2.** We begin by noticing that:

$${\bar{v}}_{x}-h_{x}=[\langle p_{x},x\rangle p_{x}-\langle x,x\rangle \frac{x}{2^{2d}}]+\left[\langle {\tilde{h}}_{x},x\rangle \frac{{\tilde{h}}_{x}}{2^{2d}}-\langle q_{x},x\rangle x\right].$$

Below we show that:

(A16) $$∥\langle p_{x},x\rangle p_{x}-\langle x,x\rangle \frac{x}{2^{2d}}∥\leq \frac{50}{2^{2d}}d^{3}\sqrt{\epsilon }{\max\{\|x\|}^{2},\|{x_{🟉}\|}^{2}\}\|x\|.$$

and

(A17) $$∥\langle q_{x},x\rangle p_{x}-\langle {\tilde{h}}_{x},x\rangle \frac{{\tilde{h}}_{x}}{2^{2d}}|\leq \frac{36}{2^{2d}}d^{4}\sqrt{\epsilon }{\max\{\|x\|}^{2},\|{x_{🟉}\|}^{2}\}\|x\|.$$

from which the thesis follows. Regarding Equation (A16) observe that:

$$\begin{array}{cc} ∥\langle p_{x},x\rangle p_{x}-\langle x,x\rangle \frac{x}{2^{2d}}∥ & =\|\langle p_{x},x\rangle [p_{x}-\frac{x}{2^{d}}]+\langle p_{x}-\frac{x}{2^{d}},\frac{x}{2^{d}}\rangle x∥\\ & \leq (∥\Lambda_{x}{x\|}^{2}+\frac{{\left\|x\right\|}^{2}}{2^{d}})∥p_{x}-\frac{x}{2^{d}}∥\\ & \leq \frac{50}{2^{2d}}d^{3}\sqrt{\epsilon }{\left\|x\right\|}^{3} \end{array}$$

where in the first inequality we used $\langle p_{x},x\rangle ={\left\|\Lambda_{x}x\right\|}^{2}$ and in the second we used Equations (A5) and (A6) of Lemma A1. Next note that:

$$\begin{array}{cc} ∥\langle q_{x},x\rangle q_{x}-\langle {\tilde{h}}_{x},x\rangle \frac{{\tilde{h}}_{x}}{2^{2d}}∥ & =\|\langle q_{x},x\rangle \left(q_{x}-\frac{{\tilde{h}}_{x}}{2^{d}}\right)+\langle q_{x}-\frac{{\tilde{h}}_{x}}{2^{d}},x\rangle \frac{{\tilde{h}}_{x}}{2^{d}}∥\\ & \leq \left(\right\|q_{x}\|+\frac{\|{\tilde{h}}_{x}\|}{2^{d}})\|x\|\|q_{x}-\frac{{\tilde{h}}_{x}}{2^{d}}∥\\ & \leq (\frac{13}{12}+1+\frac{d}{\pi })\frac{\left\|x\|\right\|x_{🟉}\|}{2^{d}}\left\|q_{x}-{\tilde{h}}_{x}\right\|\\ & \leq \frac{3}{2}d\frac{\left\|x\|\right\|x_{🟉}\|}{2^{d}}\left\|q_{x}-{\tilde{h}}_{x}\right\| \end{array}$$

where in the second inequality we have the bound (A6) and the definition of ${\tilde{h}}_{x}$. Equation (A17) is then found by appealing to Equation (A5) in Lemma A1. □

The previous lemma is now used to control the concentration of the subgradients $v_{x}$ of *f* around $h_{x}$.

**Proof of Lemma A3.** When *f* is differentiable at *x*, $\nabla f\left(x\right)={\tilde{v}}_{x}={\bar{v}}_{x}+\eta_{x}$, so that by Lemma A2 and the assumption on the noise:

(A18) $$\begin{array}{cc} \|{\tilde{v}}_{x}-h_{x}\| & \leq \|{\bar{v}}_{x}-h_{x}\left\|+\right\|\eta_{x}\|\\ \; & \leq 86\frac{d^{4}\sqrt{\epsilon }}{2^{2d}}\max(\|{x_{🟉}\|}^{2},{\left\|x\right\|}^{2})\|x\|+\frac{\omega }{2^{d}}\;\left\|x\right\|. \end{array}$$

Observe, now, that by (9), for any $x\in R^{k}$, $v_{x}\in \partial f\left(x\right)=$ conv$\left(v_{1},\ldots ,v_{t}\right)$, and therefore $v_{x}=a_{1}v_{1}+\ldots +a_{T}v_{T}$ for some $a_{1},\ldots ,a_{T}\geq 0$, $\sum_{i}a_{i}=1$. Moreover for each $v_{i}$ there exist a $w_{i}$ such that $v_{i}=\lim_{\delta \to 0^{+}}{\tilde{v}}_{x+\delta w_{i}}$. Therefore using Equation (A18), the continuity of $h_{x}$ with respect to nonzero *x* and $\sum_{i}a_{i}=1$:

$$\begin{array}{cc} \|v_{x}-h_{x}\| & \leq \sum_{i=1}^{T}a_{i}\left\|v_{i}-h_{x}\right\|\\ & \leq \sum_{i=1}^{T}a_{i}\lim_{\delta \to 0}\left\|{\tilde{v}}_{x+\delta w_{i}}-h_{x+\delta w_{i}}\right\|\\ & \leq 86\frac{d^{4}\sqrt{\epsilon }}{2^{2d}}\max(\|{x_{🟉}\|}^{2},{\left\|x\right\|}^{2})\|x\|+\frac{\omega }{2^{d}}\;\left\|x\right\|. \end{array}$$

□

The above results are now used to bound the norm of $v_{x}\in \partial f\left(x\right)$.

**Proof of Lemma A4.** Since $\epsilon <1/{\left(16\pi d^{2}\right)}^{2}$ observe that $86d^{4}\sqrt{\epsilon }\leq 2d^{2}$, therefore by the assumption on the noise level and Lemma A3 it follows that for any $v_{x}\in \partial f\left(x\right)$ and $K_{2}\leq 1$

$$∥v_{x}-h_{x}\|\leq \frac{1}{2^{2d}}\frac{5}{2}d^{2}{\max(\|x\|}^{2},\|{x_{🟉}\|}^{2})\|x\|.$$

Next observe that that since $\|{\tilde{h}}_{x}\left\|\leq d\right\|x_{🟉}\|$, we have:

$$∥h_{x}\|\leq \frac{1}{2^{2d}}\frac{5}{4}d^{2}{\max(\|x\|}^{2},\|x_{🟉}{\|}^{2})\|x\|$$

and from $\|v_{x}\left\|\leq \right\|v_{x}-h_{x}\left\|+\right\|h_{x}\|$ we obtain the thesis. □

### Appendix B.2. Supplementary Proofs for Appendix A.3

In this section we prove Lemma A5 which implies that the norm of the iterates $x_{i}$ does not increase in the region $B\left(0,R_{🟉}\right)\backslash B(0,C_{🟉}\|x_{🟉}\left\|\right)$.

**Proof of Lemma A5.** Note that the thesis is equivalent to $2\langle x,v_{x}\rangle \geq \lambda \mu {\left\|v_{x}\right\|}^{2}$. Next recall that by the WDC for any $x\in R^{k}$ and 2dϵ<1:

$$\frac{\left(1-2\epsilon d\right)}{2^{d}}{\left\|x\right\|}^{2}\leq \frac{{\left(1-2\epsilon \right)}^{d}}{2^{d}}{\left\|x\right\|}^{2}\leq {\left\|G\left(x\right)\right\|}^{2}\leq \frac{{\left(1+2\epsilon \right)}^{d}}{2^{d}}{\left\|x\right\|}^{2}\leq \frac{\left(1+4\epsilon d\right)}{2^{d}}{\left\|x\right\|}^{2}.$$

At a nonzero differentiable point $x\in R^{k}\backslash \left\{0\right\}$ with $C_{🟉}\|x_{🟉}\|<\|x\|<R_{🟉}$, then

(A19) $$\begin{array}{cc} \langle {\tilde{v}}_{x},x\rangle & \geq {\left\|G\left(x\right)\right\|}^{2}{\left(\|G\left(x\right)\right\|}^{2}-\|G\left(x_{🟉}\right){\|}^{2})-\|\Lambda_{x}^{T}H\Lambda_{x}{\left\|\|x\right\|}^{2}\\ \; & \geq \frac{{\left\|x\right\|}^{2}}{2^{2d}}\left[{\left(1-2\epsilon d\right)}^{2}{\left\|x\right\|}^{2}-\left[\left(1-2\epsilon d\right)\left(1+4\epsilon d\right)+K_{2}/d^{44}\right]{\left\|x_{🟉}\right\|}^{2}\right]\\ \; & \geq \frac{{\left\|x\right\|}^{2}}{2^{2d}}\left[{\left(1-4d\epsilon \right)\|x\|}^{2}-\left[\left(1+2d\epsilon \right)+K_{2}/d^{44}\right]{\left\|x_{🟉}\right\|}^{2}\right]. \end{array}$$

Next, by Lemma A4, the definition of the step length μ and ${\max(\|x\|}^{2},\|x_{🟉}{\|}^{2})\leq R_{🟉}^{2}$, we have:

(A20) $$\frac{\lambda \mu }{2}\|{\tilde{v}}_{x}{\|}^{2}\leq \frac{K_{3}}{2^{2d}}{\left\|x\right\|}^{4},$$

which, using $\|x\|>C_{🟉}\left\|x_{🟉}\right\|$, gives

$$\begin{array}{cc} \langle {\tilde{v}}_{x},x\rangle -\frac{\lambda \mu }{2}{\left\|{\tilde{v}}_{x}\right\|}^{2} & \geq \frac{{\left\|x\right\|}^{2}}{2^{2d}}\left[\left(1-4d\epsilon -K_{3}\right){\left\|x\right\|}^{2}-\left(1+2d\epsilon +K_{2}\right){\left\|x_{🟉}\right\|}^{2}\right]\\ & \geq \frac{{\left\|x\right\|}^{2}{\left\|x_{🟉}\right\|}^{2}}{2^{2d}}\left[\left(1-4d\epsilon -K_{3}\right)C_{🟉}-\left(1+2d\epsilon +K_{2}\right)\right]. \end{array}$$

We can then conclude by observing that by the assumptions and for small enough constants $\left(1-4d\epsilon -K_{3}\right)C_{🟉}-\left(1+2d\epsilon +K_{2}/d^{44}\right)>0$. At a non-differentiable point *x*, by the characterization of the Clarke subdifferential, we can write $v_{x}=\sum_{\ell =1}^{m}c_{\ell }v_{\ell }$ where $v_{\ell }=\lim_{\delta_{\ell }\nabla f\left(x+\delta_{\ell }w_{\ell }\right)}$ then

$$\langle x,v_{x}\rangle =\langle x,\sum_{\ell =1}^{m}c_{\ell }v_{\ell }\rangle =\sum_{\ell =1}^{m}c_{\ell }\lim_{\delta_{\ell }\to 0^{+}}\langle x+\delta_{\ell }w_{\ell },{\tilde{v}}_{x+\delta_{\ell }w_{\ell }}\rangle$$

which implies that the lower bound (A19) also holds for $\langle x,v_{x}\rangle$. Similarly Lemma A4 leads to the upper bound (A20) also for $v_{x}$, which then leads to the thesis $2\langle x,v_{x}\rangle \geq \lambda \mu {\left\|v_{x}\right\|}^{2}$. □

### Appendix B.3. Supplementary Proofs for Appendix A.4

In the next lemmas we show that $h_{x}$ is locally Lipschitz.

**Lemma** **A11.**
*For all x,y≠0*

$$∥h_{x}-h_{y}\|\leq \frac{1}{2^{2d}}\left[{2(\|x\|}^{2}+{\left\|y\right\|}^{2})+\left(d^{2}+5d^{3}\max(\frac{\left\|x\right\|}{\left\|y\right\|},\frac{\left\|y\right\|}{\left\|x\right\|})\right){\left\|x_{🟉}\right\|}^{2}\right]\left\|x-y\right\|$$

**Proof.** Note that ${\tilde{h}}_{x}=\xi \|x_{🟉}\left\|+\zeta \right\|x_{🟉}\|\hat{x}$ where ξ and ζ are defined in (A23). Then observe that by (A28) and (A29) it follows that $∥{\tilde{h}}_{x}\left\|\leq d\right\|x_{🟉}∥$. Furthermore by [16] (Lemma 18) for any nonzero $x,y\in R^{k}$ we have

(A21) $$∥{\tilde{h}}_{x}-{\tilde{h}}_{y}\|\leq \frac{9}{4}d^{2}\max\left(\frac{1}{\left\|x\right\|},\frac{1}{\left\|y\right\|}\right)\|x_{🟉}\left\|\|x-y\right\|$$

Next notice that

$$∥h_{x}-h_{y}\|\leq \frac{1}{2^{2d}}\|\langle x,x\rangle x-\langle y,y\rangle \;y\|+\frac{1}{2^{2d}}∥\langle {\tilde{h}}_{y},y\rangle \;{\tilde{h}}_{y}-\langle {\tilde{h}}_{x},x\rangle {\tilde{h}}_{x}∥,$$

where by triangle inequality the first term on the left hand side can be bounded as

$$\begin{array}{cc} \left\|\langle x,x\rangle x-\langle y,y\rangle \;y\right\| & \leq \left({\left\|x\right\|}^{2}\|x-y\|+\left|{\left\|x\right\|}^{2}-{\left\|y\right\|}^{2}\right|\;\left\|y\right\|\right)\\ & \leq \left({\left\|x\right\|}^{2}+\left\|x\|\|y\right\|+{\left\|y\right\|}^{2}\right)\left\|x-y\right\|\\ & \leq 2\left({\left\|x\right\|}^{2}+{\left\|y\right\|}^{2}\right)\left\|x-y\right\| \end{array}$$

Finally note that by from the bound (A21) we obtain:

$$\begin{array}{cc} ∥\langle {\tilde{h}}_{y},y\rangle \;{\tilde{h}}_{y}-\langle {\tilde{h}}_{x},x\rangle {\tilde{h}}_{x}∥ & \leq \|{\tilde{h}}_{x}\left\|\|x\|\right\|{\tilde{h}}_{x}-{\tilde{h}}_{y}\left\|+\right\|{\tilde{h}}_{y}\|\left|\langle {\tilde{h}}_{x},x\rangle -\langle {\tilde{h}}_{y},y\rangle \right|\\ & \leq \|{\tilde{h}}_{x}\left\|\|x\|\right\|{\tilde{h}}_{x}-{\tilde{h}}_{y}\left\|+\right\|{\tilde{h}}_{y}\left\|\right\|{\tilde{h}}_{x}\left\|\|x-y\|+\right\|{\tilde{h}}_{y}\left\|\|y\|\right\|{\tilde{h}}_{x}-{\tilde{h}}_{y}\|\\ & \leq d^{2}\|x_{🟉}{\|}^{2}\left\|x-y\|+d(\|x\|+\|y\|)\right\|x_{🟉}\left\|\right\|{\tilde{h}}_{x}-{\tilde{h}}_{y}\|\\ & \leq \left(2d^{2}+5d^{3}\max\left(\frac{\left\|x\right\|}{\left\|y\right\|},\frac{\left\|y\right\|}{\left\|x\right\|}\right)\right)\|x_{🟉}{\|}^{2}\left\|x-y\right\|. \end{array}$$

where we used

$$\left(\|x\right\|+\|y\|)\max\left(\frac{1}{\left\|x\|,\|y\right\|}\right)\leq 2\frac{\max\left(\|x\|,\|y\|\right)}{\min\left(\|x\|,\|y\|\right)}=2\max\left(\frac{\left\|x\right\|}{\left\|y\right\|},\frac{\left\|y\right\|}{\left\|x\right\|}\right).$$

□

Based on the previous lemma we can now prove that $h_{x}$ is locally Lipschitz.

**Lemma** **A12.**
*Let $x\in B\left(0,R_{🟉}\right)\backslash \left\{0\right\}$, λ∈(0,1), $\mu =K_{3}2^{2d}/\left(8d^{4}R_{🟉}^{2}\right)$ with $512\pi^{2}K_{3}<1$ and $v_{x}\in \partial f\left(x\right)$, then for $x_{\lambda }=x-\lambda \mu v_{x}$ it holds that:*

$$∥h_{x}-h_{x_{\lambda }}\|\leq \frac{7}{16}\left\|v_{x}\right\|$$

**Proof.** Consider $x\in B(0,R_{🟉})$ and observe that by Lemma A4, d≥2 and the choice of μ, for any $v_{x}\in \partial f\left(x\right)$ and any λ∈(0,1) we have $\mu \|v_{x}\|\leq \|x\|/8$. It follows that

(A22) $$\frac{7}{8}\left\|x\|\leq \right\|x_{\lambda }\|\leq \frac{9}{8}\left\|x\right\|,$$

and in particular

$$\max\left(\frac{\left\|x\right\|}{\|x_{\lambda }\|},\frac{\|x_{\lambda }\|}{\left\|x\right\|}\right)\leq \frac{8}{7}.$$

Therefore by Lemma A11 we deduce that

$$\begin{array}{cc} \|h_{x}-h_{x_{\lambda }}\| & \leq \frac{\lambda \mu }{2^{2d}}\left[{2(\|x\|}^{2}+\|x_{\lambda }{\|}^{2})+\left(d^{2}+6d^{3}\right){\left\|x_{🟉}\right\|}^{2}\right]\left\|v_{x}\right\|\\ & \leq \frac{\mu }{2^{2d}}\left(4R_{🟉}^{2}+\left(d^{2}+6d^{3}\right){\left\|x_{🟉}\right\|}^{2}\right)\left\|v_{x}\right\| \end{array}$$

where in the second inequality we used ${\max(\|x\|}^{2},\|x_{\lambda }{\|}^{2})\leq R_{🟉}^{2}$ by Proposition A1. The thesis is obtained by substituting the definition of μ and using $K_{3}\leq 1$. □

Based on the previous result we can now prove Lemma A6.

**Proof of Lemma A6.** Let $x\in B\left(0,R_{🟉}\right)\cap S_{\beta }^{c}$,

$$\begin{array}{cc} \|v_{x}\| & \geq \|h_{x}\left\|-\right\|v_{x}-h_{x}\|\\ & \geq \frac{\beta }{2^{d}}\max(\|x_{🟉}{\|}^{2}{,\|x\|}^{2})\|x\|-86\frac{d^{4}\sqrt{\epsilon }}{2^{2d}}\max(\|x_{🟉}{\|}^{2}{,\|x\|}^{2})\|x\|-\frac{\omega }{2^{d}}\left\|x\right\|\\ & \geq 6\frac{\max(\|x{\|}^{2},\|x_{🟉}{\|}^{2})\|x\|}{2^{2d}}(86d^{4}\sqrt{\epsilon }+2^{d}\omega \|x_{🟉}{\|}^{-2})\\ & \geq 6\left(\frac{\max(\|x{\|}^{2},\|x_{🟉}{\|}^{2})\|x\|}{2^{2d}}86d^{4}\sqrt{\epsilon }+\frac{\omega }{2^{d}}\left\|x\right\|\right) \end{array}$$

where we used the definition β in Equation (A7) and Lemma A3. Next take λ∈[0,1] and $x_{\lambda }=x-\lambda \mu v_{x}$ with $v_{x}\in \partial f\left(x\right)$. Then for any $v_{x_{\lambda }}\in \partial f\left(x_{\lambda }\right)$

$$\begin{array}{cc} \|v_{x}-v_{x_{\lambda }}\| & \leq \|v_{x}-h_{x}\left\|+\right\|h_{x}-h_{x_{\lambda }}\left\|+\right\|h_{x_{\lambda }}-v_{x_{\lambda }}\|\\ \; & \leq 86\frac{d^{4}\sqrt{\epsilon }}{2^{2d}}(\max(\|x_{🟉}{\|}^{2}{,\|x\|}^{2})\|x\|+\max(\|x_{🟉}{\|}^{2},\|x_{\lambda }{\|}^{2})\|x_{\lambda }\|)\\ \; & \;+\frac{\omega }{2^{d}}\;(\|x\|+\|x_{\lambda }\left\|\right)+\frac{7}{16}\left\|v_{x}\right\|\\ \; & \leq 86\frac{d^{4}\sqrt{\epsilon }}{2^{2d}}\left(1+{\left(\frac{9}{8}\right)}^{3}\right)\max(\|x_{🟉}{\|}^{2}{,\|x\|}^{2})\|x\|+\left(1+\frac{9}{8}\right)\frac{\omega }{2^{d}}\left\|x\right\|+\frac{7}{16}\|v_{x}\left\|\right|\\ \; & \leq 3\left(86\frac{d^{4}\sqrt{\epsilon }}{2^{2d}}\max(\|x_{🟉}{\|}^{2}{,\|x\|}^{2})\|x\|+\frac{\omega }{2^{d}}\left\|x\right\|\right)+\frac{7}{16}\left\|v_{x}\right\|\\ \; & \leq \left(\frac{1}{2}+\frac{7}{16}\right)\left\|v_{x}\right\| \end{array}$$

where in the first inequality we used Lemma A3 and Lemma A12, in the second inequality (A22) and in the last one (A8). □

### Appendix B.4. Supplementary Proofs for Appendix A.5

Below we prove that the region of $R^{k}$ where we cannot control the norm of the vector field $h_{x}$ is contained in two balls around $x_{🟉}$ and $-\rho_{d}x_{🟉}$.

We prove Lemma A7 by showing the following.

**Lemma** **A13.**
*Suppose $8\pi d^{6}\sqrt{\beta }\leq 1$. Define:*

$$\rho_{d}:=\sum_{i=0}^{d-1}\frac{\sin{\overset{ˇ}{\theta }}_{i}}{\pi }(\prod_{j=i+1}^{d-1}\frac{\pi -{\overset{ˇ}{\theta }}_{j}}{\pi })$$

*where ${\overset{ˇ}{\theta }}_{0}=\pi$ and ${\overset{ˇ}{\theta }}_{i}=g\left({\overset{ˇ}{\theta }}_{i-1}\right)$. If $x\in S_{\beta }$, then we have either:*

$$|{\bar{\theta }}_{0}|\leq 32d^{4}{\pi \beta \;\mathrm{and}\;|\|x\|}^{2}-\|x_{🟉}{\|}^{2}|\leq 258\pi \beta d^{6}\left\|x_{🟉}\right\|$$

*or*

$$|{\bar{\theta }}_{0}-\pi |\leq 8\pi d^{4}\sqrt{\beta }{\;\mathrm{and}\;|\|x\|}^{2}-\rho_{d}^{2}\|x_{🟉}{\|}^{2}|\leq 281\pi^{2}\sqrt{\beta }d^{10}\left\|x_{🟉}\right\|.$$

*In particular, we have:*

$$S_{\beta }\subset B(x_{🟉},R_{1}\beta d^{10}\|x_{🟉}\|)\cup B(-\rho_{d}x_{🟉},R_{2}\sqrt{\beta }d^{10}\|x_{🟉}\left\|\right)$$

*where $R_{1},R_{2}$ are numerical constants and $\rho_{d}\to 1$ as d→∞.*

**Proof of Lemma A13.** Without loss of generality, let $x_{🟉}=e_{1}$ and $x=r\cos{\bar{\theta }}_{0}\cdot e_{1}+r\sin{\bar{\theta }}_{0}\cdot e_{2}$, for some ${\bar{\theta }}_{0}\in \left[0,\pi \right]$, and r≥0. Recall that we call $\hat{x}=x/\left\|x\right\|$ and ${\hat{x}}_{🟉}=x_{🟉}/\left\|x_{🟉}\right\|$. We then introduce the following notation:

(A23) $$\xi =\prod_{i=0}^{d-1}\frac{\pi -{\bar{\theta }}_{i}}{\pi },\;\zeta =\sum_{i=0}^{d-1}\frac{\sin{\bar{\theta }}_{i}}{\pi }\prod_{j=i+1}^{d-1}\frac{\pi -{\bar{\theta }}_{j}}{\pi },\;r=\left\|x\right\|,\;R=\max\left(r^{2},1\right),$$

where $\theta_{i}=g\left({\bar{\theta }}_{i-1}\right)$ with *g* as in (A1), and observe that ${\tilde{h}}_{x}=\left(\xi {\hat{x}}_{🟉}+\zeta \hat{x}\right)$. Let $\alpha :=\langle {\tilde{h}}_{x},\hat{x}\rangle$, then we can write:

$$\begin{array}{c} h_{x}=\frac{1}{2^{2d}}\left[\langle x,x\rangle x-\langle {\tilde{h}}_{x},x\rangle {\tilde{h}}_{x}\right]=\frac{r}{2^{2d}}\left[r^{2}\hat{x}-\alpha \left(\xi {\hat{x}}_{🟉}+\zeta \hat{x}\right)\right]. \end{array}$$

Using the definition of $\hat{x}$ and ${\hat{x}}_{🟉}$ we obtain:

$$\frac{2^{2d}h_{x}}{r}=\left[\left(r^{2}-\alpha \;\zeta \right)\cos{\bar{\theta }}_{0}-\alpha \;\xi \right]\cdot e_{1}+\left[r^{2}-\alpha \zeta \right]\sin{\bar{\theta }}_{0}\cdot e_{2},$$

and conclude that since $x\in S_{\beta }$, then:

(A24) $$\begin{array}{cc} |\left(r^{2}-\alpha \;\zeta \right)\cos{\bar{\theta }}_{0}-\alpha \;\xi | & \leq \beta R \end{array}$$

(A25) $$\begin{array}{c} |\left[r^{2}-\alpha \zeta \right]\sin{\bar{\theta }}_{0}|\leq \beta R. \end{array}$$

We now list some bounds that will be useful in the subsequent analysis. We have:

(A26) $$\begin{array}{cc} {\bar{\theta }}_{i} & \leq {\bar{\theta }}_{i-1}\;\;\mathrm{for}\;\;i\geq 1 \end{array}$$

(A27) $$\begin{array}{cc} {\bar{\theta }}_{i} & \leq \cos^{-1}\left(1/\pi \right)\;\;\mathrm{for}\;\;i\geq 2 \end{array}$$

(A28) $$\begin{array}{cc} \left|\xi \right| & \leq 1 \end{array}$$

(A29) $$\begin{array}{cc} \left|\zeta \right| & \leq \frac{d}{\pi }\sin{\bar{\theta }}_{0} \end{array}$$

(A30) $$\begin{array}{cc} \xi & \geq \frac{\pi -{\bar{\theta }}_{0}}{\pi }d^{-3} \end{array}$$

(A31) $$\begin{array}{cc} {\overset{ˇ}{\theta }}_{i} & \leq \frac{3\pi }{i+3}\;\;\mathrm{for}\;\;i\geq 0 \end{array}$$

(A32) $$\begin{array}{cc} {\overset{ˇ}{\theta }}_{i} & \geq \frac{\pi }{i+1}\;\;\mathrm{for}\;\;i\geq 0 \end{array}$$

(A33) $$\begin{array}{cc} {\bar{\theta }}_{0} & =\pi +O_{1}\left(\delta \right)\Rightarrow \left|\xi \right|\leq \frac{\delta }{\pi } \end{array}$$

(A34) $$\begin{array}{cc} {\bar{\theta }}_{0} & =\pi +O_{1}\left(\delta \right)\Rightarrow \zeta =\rho_{d}+O_{1}\left(3d^{3}\delta \right)\;\mathrm{if}\;\frac{d^{2}\delta }{\pi }\leq 1 \end{array}$$

(A35) $$\begin{array}{cc} 1/\pi & \leq \alpha \leq 1. \end{array}$$

The identities (A26) through (A34) can be found in Lemma 16 of [16], while the identity (A35) follows by noticing that $\alpha =\xi \cos{\bar{\theta }}_{0}+\zeta =\cos\theta_{d}$ and using (A27) together with d≥2.

**Bound on *R*.** We now show that if $x\in S_{\beta }$, then $r^{2}\leq 4d$ and therefore R≤4d. If $r^{2}\leq 1$, then the claim is trivial. Take $r^{2}>1$, then note that either $|\sin{\bar{\theta }}_{0}|\geq 1/\sqrt{2}$ or $|\cos{\bar{\theta }}_{0}|\geq 1/\sqrt{2}$ must hold. If $|\sin{\bar{\theta }}_{0}|\geq 1/\sqrt{2}$ then from (A25) it follows that $r^{2}-\alpha \zeta \leq \sqrt{2}\beta R=\sqrt{2}\beta r^{2}$ which implies:

$$r^{2}\leq \frac{\alpha \;\zeta }{1-\sqrt{2}\beta }\leq \frac{1}{\left(1-\sqrt{2}\beta \right)}\frac{d}{\pi }\leq \frac{d}{2}$$

using (A29) and (A35) in the second inequality and β<1/4 in the third. Next take $|\cos{\bar{\theta }}_{0}|\geq 1/\sqrt{2}$, then (A24) implies $|r^{2}-\alpha \zeta |\leq \sqrt{2}\left(\beta r^{2}+\alpha \xi \right)$ which in turn results in:

$$r^{2}\leq \frac{\alpha (\zeta +\sqrt{2}\xi )}{1-\sqrt{2}\beta }\leq 4d$$

using (A28), (A29), (A35) and β<1/4. In conclusion if $x\in S_{\beta }$ then $r^{2}\leq 4d\Rightarrow R\leq 4d$.

**Bounds on ${\bar{\theta }}_{0}$.** We proceed by showing that we only have to analyze the small angle case ${\bar{\theta }}_{0}\approx 0$ and the large angle case ${\bar{\theta }}_{0}\approx \pi$. At least one of the following three cases must hold:

(1) $\sin{\bar{\theta }}_{0}\leq 16\beta \pi d^{4}$: Then we have $\bar{\theta }=O_{1}\left(32\pi \beta \pi d^{4}\right)$ or $\bar{\theta }=\pi +O_{1}\left(32\pi \beta \pi d^{4}\right)$ as $32\pi \beta \pi d^{4}<\;1$.
(2) $|r^{2}-\alpha \zeta |<\sqrt{\beta }R$: Then (A24), (A35) and β<1 yield $\left|\xi \right|\leq 2\sqrt{\beta }\pi R$. Using (A30), we then get $\bar{\theta }\;=\;\pi \;+\;O_{1}\left(2\sqrt{\beta }\pi^{2}d^{3}R\right)$.
(3) $\sin{\bar{\theta }}_{0}>16\beta \pi d^{4}$ and $|r^{2}-\alpha \zeta |\geq \sqrt{\beta }R$: Then (A27) gives $|r^{2}-\alpha \zeta |\leq \beta M/\sin{\bar{\theta }}_{0}$ which used with (A24) leads to:
  $$\left|\alpha \xi |\leq \beta R+\right|r^{2}-\alpha \zeta |\leq \beta R+\frac{\beta R}{\sin{\bar{\theta }}_{0}}\leq 2\frac{\beta R}{\sin{\bar{\theta }}_{0}}.$$
  Then using (A35), the assumption on $\sin{\bar{\theta }}_{0}$ and R≤4d we obtain $\xi \leq d^{-3}/2$. The latter together with (A30) leads to ${\bar{\theta }}_{0}\geq \pi /2$. Finally as $|r^{2}-\alpha \zeta |\geq \sqrt{\beta }R$ then (A25) leads to $|\sin{\bar{\theta }}_{0}|\leq \sqrt{\beta }$. Therefore as ${\bar{\theta }}_{0}\geq \pi /2$ and β<1, we can conclude that ${\bar{\theta }}_{0}=\pi +O_{1}\left(2\sqrt{\beta }\right)$.

Inspecting the three cases, and recalling that R≤4d, we can see that it suffices to analyze the small angle case ${\bar{\theta }}_{0}=O_{1}\left(32d^{4}\pi \beta \right)$ and the large angle case $\bar{\theta }=\pi +O_{1}\left(8\sqrt{\beta }\pi^{2}d^{4}\right)$.

**Small angle case.** We assume ${\bar{\theta }}_{0}=O_{1}\left(\delta \right)$ with $\delta =32d^{4}\pi \beta$ and show that ${\left\|x\right\|}^{2}\approx {\left\|x_{🟉}\right\|}^{2}$. We begin collecting some bounds. Since ${\bar{\theta }}_{i}\leq {\bar{\theta }}_{0}\leq \delta$, then $1\geq \xi \geq {\left(1-\delta /\pi \right)}^{d}\geq 1+O_{1}\left(2d\delta /\pi \right)$ assuming δd/π≤1/2, which holds true since $64d^{5}\beta <1$. Moreover from (A29) we have $\zeta =O_{1}\left(d\delta /\pi \right)$. Finally observe that $\cos{\bar{\theta }}_{0}=1+O_{1}\left({\bar{\theta }}_{0}^{2}/2\right)=1+O_{1}\left(\delta /2\right)$ for δ<1. We then have $\alpha =1+O_{1}\left(2d\delta \right)$ so that $\alpha \zeta =O_{1}\left(d^{2}\delta \right)$ and $\alpha \xi =1+O_{1}\left(4d^{2}\delta \right)$. We can therefore rewrite (A24) as:

$$\left(r^{2}+O_{1}\left(d^{2}\delta \right)\right)\left(1+O_{1}\left(\delta /2\right)\right)-\left(1+O_{1}\left(4d^{2}\delta \right)\right)=O_{1}\left(\beta R\right).$$

Using the bound $r^{2}\leq R\leq 4d$ and the definition of δ, we obtain:

(A36) $$\begin{array}{cc} r^{2}-1 & =O_{1}\left(\frac{\delta r^{2}}{2}+d^{2}\delta +\frac{d^{2}\delta^{2}}{2}+4d^{2}\delta +4d\beta \right)\\ \; & =O_{1}\left(8d^{2}\delta +4d\beta \right)\\ \; & =O_{1}\left(258\pi d^{6}\beta \right) \end{array}$$

**Large angle case.** Here we assume $\bar{\theta }=\pi +O_{1}\left(\delta \right)$ with $\delta =8\sqrt{\beta }\pi^{2}d^{4}$ and show that it must be ${\left\|x\right\|}^{2}\approx \rho_{d}^{2}{\left\|x_{🟉}\right\|}^{2}$. From (A33) we know that $\xi =O_{1}\left(\delta /\pi \right)$, while from (A34) we know that $\zeta =\rho_{d}+O_{1}\left(3d^{3}\delta \right)$ as long as $8\sqrt{\beta }\pi d^{6}\leq 1$. Moreover for large angles and δ<1, it holds $\cos{\bar{\theta }}_{0}=-1+O_{1}\left({\left({\bar{\theta }}_{0}-\pi \right)}^{2}/2\right)=-1+O_{1}\left(\delta^{2}/2\right)$. These bounds lead to:

$$\begin{array}{cc} \alpha & =\xi \cos{\bar{\theta }}_{0}+\zeta \\ & =\rho_{d}+O_{1}\left(\frac{\delta }{\pi }+\frac{\delta^{3}}{2\pi }+3d^{3}\delta \right)\\ & =\rho_{d}+O_{1}\left(4d^{3}\delta \right), \end{array}$$

and using $\rho_{d}\leq d$:

$$\begin{array}{cc} \alpha \zeta & =\rho_{d}^{2}+O_{1}\left(4d^{3}\delta \rho_{d}+3d^{3}\delta \rho_{d}+12d^{6}\delta \right)=\rho_{d}^{2}+O_{1}\left(20d^{6}\delta \right),\\ \alpha \xi & =O_{1}\left(\frac{\delta }{\pi }\rho_{d}+4\frac{d^{3}\delta^{2}}{\pi }\right)=O_{1}\left(2d^{3}\delta \right). \end{array}$$

Then recall that (A24) is equivalent to $\left(r^{2}-\alpha \zeta \right)\cos{\bar{\theta }}_{0}-\alpha \xi =O_{1}\left(4\beta d\right)$, that is:

$$\left(r^{2}-\rho_{d}^{2}+O_{1}\left(20d^{6}\delta \right)\right)\left(1+O_{1}\left(\delta^{2}/2\right)\right)+O_{1}\left(2d^{3}\delta \right)=O_{1}\left(4\beta d\right)$$

and in particular:

(A37) $$\begin{array}{cc} r^{2}-\rho_{d}^{2} & =O_{1}\left(20d^{6}\delta +10d^{6}\delta^{3}+\frac{\rho_{d}\delta^{2}}{2}+\frac{r^{2}\delta^{2}}{2}+2d^{3}\delta +4\beta d\right)\\ \; & =O_{1}\left(35d^{6}\delta +4\beta d\right)\\ \; & =O_{1}\left(281\pi^{2}\sqrt{\beta }d^{10}\right) \end{array}$$

where we used $\rho_{d}\leq d$, the definition of δ and δ<1.

**Controlling the distance.** We have shown that it is either ${\bar{\theta }}_{0}\approx 0$ and ${\left\|x\right\|}^{2}\approx {\left\|x_{🟉}\right\|}^{2}$ or ${\bar{\theta }}_{0}\approx \pi$ and ${\left\|x\right\|}^{2}\approx \rho_{d}^{2}{\left\|x_{🟉}\right\|}^{2}$. We can therefore conclude that it must be either $x\approx x_{🟉}$ or $x\approx -\rho_{d}x_{🟉}$. Observe that if a two dimensional point is known to have magnitude within Δr of some *r* and is known to be within an angle Δθ from 0, then its Euclidean distance to the point of coordinates (r,0) is no more that Δr+(r+Δr)Δθ. Similarly we can write:

(A38) $$\|x-x_{🟉}\left\|\leq |\|x\|-\right\|x_{🟉}\left\|\right|+\left(\right\|x_{🟉}\left\|+|\|x\|-\right\|x_{🟉}\left\||\right){\bar{\theta }}_{0}.$$

In the small angle case, by (A36), (A38), and $\|x_{🟉}\left\|\;|\|x\|-\right\|x_{🟉}\left\|\right|\leq {\left|\|x\right\|}^{2}-\|x_{🟉}{\|}^{2}|$, we have:

$$\|x-x_{🟉}\|\leq 258\pi d^{6}\beta +\left(1+258\pi d^{6}\beta \right)\;32d^{4}\pi \beta \leq 550\;\pi d^{10}\beta .$$

Next we notice that $\rho_{2}=1/\pi$ and $\rho_{d}\geq \rho_{d-1}$ as follows from the definition and (A31), (A32). Then considering the large angle case and using (A37) we have:

$$\left|\|x\right\|-\rho_{d}|\leq \frac{281\pi^{2}\sqrt{\beta }d^{10}}{\left\|x\right\|+\rho_{d}}\leq 281\pi^{3}\sqrt{\beta }d^{10}.$$

The latter, together with (A38), yields:

$$\begin{array}{cc} \|x+\rho_{d}x_{🟉}\| & \leq \left|\|x\right\|-\rho_{d}|+(\rho_{d}+\left|\|x\right\|-\rho_{d}\left|\right)\left(\pi -{\bar{\theta }}_{0}\right)\\ & \leq 281\pi^{3}\sqrt{\beta }d^{10}+\left(d+281\pi^{3}\sqrt{\beta }d^{10}\right)8\sqrt{\beta }\pi^{2}d^{4}\\ & \leq 284\pi^{3}\sqrt{\beta }d^{10} \end{array}$$

where in the second inequality we have used $\rho_{d}\leq d$ and in the third $8\sqrt{\beta }\pi d^{6}\leq 1$. We conclude by noticing that $\rho_{d}\to 1$ as d→1 as shown in [16] (Lemma 16). □

We next will show that the values of the loss function in a neighborhood of $x_{🟉}$ are strictly smaller that those in a neighborhood of $-\rho_{d}x_{🟉}$.

Recall that ${f\left(x\right):=1/4\|G\left(x\right)G\left(x\right)}^{T}-G\left(x_{🟉}\right)G{\left(x_{🟉}\right)}^{T}{-H\|}_{F}^{2}$, we next define the following loss functions:

$$\begin{array}{cc} f_{0}\left(x\right) & :=\frac{1}{4}{\left\|G\left(x\right)G{\left(x\right)}^{T}-G\left(x_{🟉}\right)G{\left(x_{🟉}\right)}^{T}\right\|}_{F}^{2},\\ f_{H}\left(x\right) & :=f_{0}\left(x\right)-\frac{1}{2}{\langle G\left(x\right)G{\left(x\right)}^{T}-G\left(x_{🟉}\right)G{\left(x_{🟉}\right)}^{T},H\rangle }_{F},\\ f_{E}\left(x\right) & :=\frac{1}{2^{2d+2}}\left({\left\|x\right\|}^{4}+{\left\|x_{🟉}\right\|}^{4}-2{\langle x,{\tilde{h}}_{x}\rangle }^{2}\right). \end{array}$$

In particular notice that $f\left(x\right)=f_{H}\left(x\right)+1/4{\left\|H\right\|}_{F}^{2}$. Below we show that assuming the WDC is satisfied, $f_{0}\left(x\right)$ concentrates around $f_{E}\left(x\right)$.

**Lemma** **A14.**
*Suppose that d≥2 and the WDC holds with $\epsilon <1/{\left(16\pi d^{2}\right)}^{2}$, then for all nonzero $x,x_{🟉}\in R^{k}$*

$$|f_{0}\left(x\right)-f_{E}\left(x\right)|\leq \frac{16}{2^{2d}}{\left(\|x\right\|}^{4}+\|x_{🟉}{\|}^{4})d^{4}\sqrt{\epsilon }$$

**Proof.** Observe that:

$$\begin{array}{cc} |f_{0}\left(x\right)-f_{E}\left(x\right)| & \leq \frac{1}{4}{\left|\|G\left(x\right)\right\|}^{4}-\frac{1}{2^{2d}}{\left\|x\right\|}^{4}|\\ & +\frac{1}{4}|\|G\left(x_{🟉}\right){\|}^{4}-\frac{1}{2^{2d}}\|x_{🟉}{\|}^{4}|\\ & +\frac{1}{2}\left|{\langle G\left(x\right),G\left(x_{🟉}\right)\rangle }^{2}-\frac{1}{2^{2d}}{\langle x,{\tilde{h}}_{x}\rangle }^{2}\right|. \end{array}$$

We analyze each term separately. The first term can be bounded as:

$$\begin{array}{cc} \frac{1}{4}{\left|\|G\left(x\right)\right\|}^{4}-\frac{1}{2^{2d}}{\left\|x\right\|}^{4}| & =\frac{1}{4}{\left|\|G\left(x\right)\right\|}^{2}+\frac{1}{2^{d}}{\left\|x\right\|}^{2}{\left|\;\;|\|G\left(x\right)\right\|}^{2}-\frac{1}{2^{d}}{\left\|x\right\|}^{2}|\\ \; & \leq \frac{1}{4}\frac{1}{2^{d}}(\frac{13}{12}+1){\left\|x\right\|}^{2}{\;\;|\|G\left(x\right)\|}^{2}-\frac{1}{2^{d}}{\left\|x\right\|}^{2}|\\ \; & \leq \frac{1}{4}\frac{1}{2^{d}}(\frac{13}{12}+1){\left\|x\right\|}^{2}\;\;24\frac{d^{3}\sqrt{\epsilon }}{2^{d}}{\left\|x\right\|}^{2}\\ \; & \leq \frac{1}{2^{2d}}13d^{3}\;\sqrt{\epsilon }{\left\|x\right\|}^{4} \end{array}$$

where in the first inequality we used (A6) and in the second inequality (A5). Similarly we can bound the second term:

$$\frac{1}{4}|\|G\left(x_{🟉}\right){\|}^{4}-\frac{1}{2^{2d}}\|x_{🟉}{\|}^{4}|\leq \frac{1}{2^{2d}}13d^{3}\;\sqrt{\epsilon }{\left\|x_{🟉}\right\|}^{4}.$$

We next note that $\|{\tilde{h}}_{x}\left\|\leq \left(1+d/\pi \right)\right\|x_{🟉}\|$ and therefore from (A6) and d≥2 we obtain:

$$|\|G\left(x\right)\|\|G\left(x_{🟉}\right)\left\|+\|x\right\|\frac{\|{\tilde{h}}_{x}\|}{2^{d}}|\leq \frac{1}{2^{d}}(\frac{13}{12}+1+\frac{d}{\pi })\left\|x\|\right\|x_{🟉}\|\leq \frac{1}{2^{d}}\frac{3}{2}d\|x\|\|x_{🟉}∥$$

We can then conclude that:

$$\begin{array}{cc} \frac{1}{2}\left|{\langle G\left(x\right),G\left(x_{🟉}\right)\rangle }^{2}-{\langle x,\frac{{\tilde{h}}_{x}}{2^{d}}\rangle }^{2}\right| & \leq \frac{1}{2}|\langle x,\Lambda_{x}^{T}\Lambda_{x_{🟉}}x_{🟉}-{\tilde{h}}_{x}\rangle |\;\;|\|G\left(x\right)\|\|G\left(x_{🟉}\right)\left\|+\|x\|\right\|{\tilde{h}}_{x}\left\|\right|\\ & \leq \frac{1}{2}\|x\|24\frac{d^{3}\sqrt{\epsilon }}{2^{d}}\|x_{🟉}\|\;\;\frac{1}{2^{d}}\frac{3}{2}d\;\|x\|\|x_{🟉}∥\\ & \leq \frac{9}{2^{2d}}d^{4}\sqrt{\epsilon }\left(\right\|x_{🟉}{\|}^{4}+{\left\|x\right\|}^{4}) \end{array}.$$

□

We next consider the loss $f_{E}$ and show that in a neighborhood $-\rho_{d}x_{🟉}$, this loss function has larger values than in a neighborhood of $x_{🟉}$.

**Lemma** **A15.**
*Fix $0<a\leq 1/(2\pi^{3}d^{3})$ and $\phi_{d}\in \left[\rho_{d},1\right]$ then:*

$$\begin{array}{cc} f_{E}\left(x\right) & \leq \frac{1}{2^{2d+2}}\|x_{🟉}{\|}^{4}+\frac{1}{2^{2d+2}}[{\left(a+\phi_{d}\right)}^{4}-2\phi_{d}^{2}+2\pi da]\|x_{🟉}{\|}^{4}\;\forall x\in B(\phi_{d}x_{🟉},a\|x_{🟉}\left\|\right)\;\mathrm{and}\\ f_{E}\left(x\right) & \geq \frac{1}{2^{2d+2}}\|x_{🟉}{\|}^{4}+\frac{1}{2^{2d+2}}[{\left(a-\phi_{d}\right)}^{4}-2\rho_{d}^{2}\phi_{d}^{2}-40\pi d^{3}a]\|x_{🟉}{\|}^{4}\;\forall x\in B(-\phi_{d}x_{🟉},a\|x_{🟉}\left\|\right). \end{array}$$

**Proof.** Let $x\in B(\phi_{d}x_{🟉},a\|x_{🟉}\|)$ then observe that $0\leq {\bar{\theta }}_{i}\leq {\bar{\theta }}_{0}\leq \pi a/2\phi_{d}$ and $\left(\phi_{d}-a\right)\|x_{🟉}\|\leq \|x\|\leq \left(a+\phi_{d}\right)\left\|x_{🟉}\right\|$. Then observe that:

$$\begin{array}{cc} \langle x,\frac{{\tilde{h}}_{d}}{2^{d}}\rangle & =\frac{1}{2^{d}}(\prod_{i=0}^{d-1}\frac{\pi -{\bar{\theta }}_{i}}{\pi })\|x_{🟉}\|\|x\|\cos{\bar{\theta }}_{0}+\frac{1}{2^{d}}\sum_{i=0}^{d-1}\frac{\sin{\bar{\theta }}_{i}}{\pi }\prod_{j=i+1}^{d-1}\frac{\pi -{\bar{\theta }}_{j}}{\pi }\;\|x_{🟉}\left\|\|x\right\|\\ & \geq \frac{1}{2^{d}}(\prod_{i=0}^{d-1}\frac{\pi -\frac{\pi a}{2\phi_{d}}}{\pi })\;\left(\phi_{d}-a\right){\left\|x_{🟉}\right\|}^{2}(1-\frac{\pi^{2}a^{2}}{8\phi_{d}^{2}})\\ & \geq \frac{1}{2^{d}}(1-\frac{da}{\phi_{d}})\left(\phi_{d}-a\right)(1-\frac{\pi^{2}a^{2}}{8\phi_{d}^{2}}){\left\|x_{🟉}\right\|}^{2}. \end{array}$$

using $\cos\theta \geq 1-\theta^{2}/2$ and ${\left(1-x\right)}^{d}\geq \left(1-2dx\right)$ as long as 0≤x≤1. We can therefore write:

$$\begin{array}{cc} f_{E}\left(x\right)-\frac{\|x_{🟉}{\|}^{4}}{2^{2d+2}} & \leq \frac{1}{2^{2d+2}}{\left\|x\right\|}^{4}-\frac{1}{2^{2d+1}}{\left(1-\frac{da}{\phi_{d}}\right)}^{2}{\left(\phi_{d}-a\right)}^{2}{\left(1-\frac{\pi^{2}a^{2}}{8\phi_{d}^{2}}\right)}^{2}{\left\|x_{🟉}\right\|}^{4}\\ & \leq \frac{1}{2^{2d+2}}[{\left(\phi_{d}+a\right)}^{4}-2(1-2\frac{da}{\phi_{d}}){\left(\phi_{d}-a\right)}^{2}(1-\frac{\pi^{2}a^{2}}{4\phi_{d}^{2}})]{\left\|x_{🟉}\right\|}^{4} \end{array}$$

where in the second inequality we used ${\left(1-x\right)}^{2}\geq 1-2x\;$ for all x∈R. We then observe that:

$$\begin{array}{cc} \left(1-2\frac{da}{\phi_{d}}){\left(\phi_{d}-a\right)}^{2}(1-\frac{\pi^{2}a^{2}}{4\phi_{d}^{2}}\right) & \geq (1-\frac{\pi^{2}a^{2}}{4\phi_{d}^{2}}-\frac{2ad}{\phi_{d}})\phi_{d}^{2}+a\left(a-2\phi_{d}\right)(1-2\frac{da}{\phi_{d}})(1-\frac{\pi^{2}a^{2}}{4\phi_{d}^{2}})\\ & \geq \phi_{d}^{2}-a(\frac{1}{2\pi d^{3}}+2d\phi_{d})+a\left(a-2\phi_{d}\right)(1-2\frac{da}{\phi_{d}})(1-\frac{\pi^{2}a^{2}}{4\phi_{d}^{2}})\\ & \geq \phi_{d}^{2}-a(\frac{1}{2\pi d^{3}}+2d\phi_{d}+2\phi_{d})\\ & \geq \phi_{d}^{2}-\pi da, \end{array}$$

where in the second inequality we have used $\pi^{3}d^{3}a\leq 2$ and in the last one d≥2 and $\phi_{d}\leq 1$. We can then conclude that:

$$f_{E}\left(x\right)-\frac{\|x_{🟉}{\|}^{4}}{2^{2d+2}}\leq \frac{1}{2^{2d+2}}\left[{\left(\phi_{d}+a\right)}^{4}-2\left(\phi_{d}^{2}-\pi da\right)\right]{\left\|x_{🟉}\right\|}^{4}$$

We next take $x\in B(-\phi_{d}x_{🟉},a\|x_{🟉}\|)$ which implies $0\leq \pi -{\bar{\theta }}_{0}\leq \pi^{2}a/2=:\delta$ and $\left\|x\right\|\leq \left(a+\phi_{d}\right)\left\|x_{🟉}\right\|$. We then note that for ξ and ζ as defined in (A23) we have:

$$\begin{array}{cc} |x^{T}{\tilde{h}}_{x}{|}^{2} & \leq \left(|\xi \right|+{\left|\zeta |\right)}^{2}{\left(a+\phi_{d}\right)}^{2}{\left\|x_{🟉}\right\|}^{4}\\ & \leq {\left(\frac{\delta }{\pi }+3d^{3}\delta +\rho_{d}\right)}^{2}{\left(a+\phi_{d}\right)}^{2}{\left\|x_{🟉}\right\|}^{4}\\ & \leq {\left(\frac{\pi^{3}d^{3}}{2}a+\rho_{d}\right)}^{2}{\left(a+\phi \right)}^{2}{\left\|x_{🟉}\right\|}^{4}\\ & \leq \left(2\pi^{3}d^{3}a+\rho_{d}^{2}\right){\left(a+\phi_{d}\right)}^{2}{\left\|x_{🟉}\right\|}^{4}\\ & \leq 20\pi d^{3}a+\rho_{d}^{2}\phi_{d}^{2} \end{array}$$

where the second inequality is due to (A33) and (A34), the rest from d≥2, $\rho_{d}\leq \phi_{d}\leq 1$ and $2\pi^{3}d^{3}a\leq 1$. Finally using $\left(\phi_{d}-a\right)\|x_{🟉}\left\|\leq \|x\right\|$, we can then conclude that:

$$f_{E}\left(x\right)-\frac{\|x_{🟉}{\|}^{4}}{2^{2d+2}}\geq \frac{1}{2^{2d+2}}\left[{\left(\phi_{d}-a\right)}^{4}-2\left(20\pi d^{3}a+\rho_{d}^{2}\phi_{d}^{2}\right)\right]{\left\|x_{🟉}\right\|}^{4}.$$

□

The above two lemmas are now used to prove Lemma A8.

**Proof of Proposition A8.** Let $x\in B(\pm \phi_{d}\;x_{🟉},\varphi \|x_{🟉}\|)$ for a 0<φ<1 that will be specified below, and observe that by the assumptions on the noise:

$$\begin{array}{cc} |{\langle G\left(x\right)G{\left(x\right)}^{T}-G\left(x_{🟉}\right)G{\left(x_{🟉}\right)}^{T},H\rangle }_{F}| & {\leq |G\left(x\right)}^{T}HG\left(x\right)|+|G{\left(x_{🟉}\right)}^{T}HG\left(x_{🟉}\right)|\\ & \leq \frac{\omega }{2^{d}}{\left(\|x\right\|}^{2}+\|x_{🟉}{\|}^{2})\\ & \leq \frac{\omega }{2^{d}}\left({\left(\phi_{d}+\varphi \right)}^{2}+1\right){\left\|x_{🟉}\right\|}^{2}, \end{array}$$

and therefore by Lemma A14:

$$\begin{array}{cc} |f_{0}\left(x\right)-f_{E}\left(x\right)| & +\frac{1}{2}\left|{\langle G\left(x\right)G{\left(x\right)}^{T}-G\left(x_{🟉}\right)G{\left(x_{🟉}\right)}^{T},H\rangle }_{F}\right|\leq \\ & \leq \frac{16}{2^{2d}}\left({\left(\phi_{d}+\varphi \right)}^{4}+1\right)\|x_{🟉}{\|}^{4}d^{4}\sqrt{\epsilon }+\frac{\omega }{2^{d}}\left({\left(\phi_{d}+\varphi \right)}^{2}+1\right){\left\|x_{🟉}\right\|}^{2}\\ & \leq \frac{272}{2^{2d}}\|x_{🟉}{\|}^{4}d^{4}\sqrt{\epsilon }+\frac{\omega }{2^{d}}\left({\left(\phi_{d}+\varphi \right)}^{2}+1\right){\left\|x_{🟉}\right\|}^{2} \end{array}$$

We next take φ=ϵ and $x\in B(\phi_{d}\;x_{🟉},\varphi \|x_{🟉}\|)$, so that by Lemma A15 and the assumption $2^{d}d^{44}w\leq K_{2}{\left\|x_{🟉}\right\|}^{2}$, we have:

$$\begin{array}{cc} f_{H}\left(x\right) & \leq f_{E}\left(x\right)+|f_{0}\left(x\right)-f_{E}\left(x\right)|+\frac{1}{2}\left|{\langle G\left(x\right)G{\left(x\right)}^{T}-G\left(x_{🟉}\right)G{\left(x_{🟉}\right)}^{T},H\rangle }_{F}\right|\\ & \leq \frac{1}{2^{2d+2}}\left[1+{\left(\epsilon +\phi_{d}\right)}^{4}-2\phi_{d}^{2}+2\pi d\epsilon \right]\|x_{🟉}{\|}^{4}+272d^{4}\sqrt{\epsilon }\|x_{🟉}{\|}^{4}+\frac{\omega }{2^{d+1}}\left(2+2\epsilon +\epsilon^{2}\right){\left\|x_{🟉}\right\|}^{2}\\ & \leq \frac{1}{2^{2d+2}}\left[1-2\phi_{d}^{2}+{\left(\epsilon +\phi_{d}\right)}^{4}\right]\|x_{🟉}{\|}^{4}+\frac{1}{2^{2d}}\left(\frac{3}{2}2^{d}{\left\|x_{🟉}\right\|}^{-2}\omega +\frac{\pi d}{2}+272d^{4}\right)\sqrt{\epsilon }\|x_{🟉}{\|}^{4}+\frac{\omega }{2^{d}}{\left\|x_{🟉}\right\|}^{2}\\ & \leq \frac{1}{2^{2d+2}}\left[1-2\phi_{d}^{2}+{\left(\epsilon +\phi_{d}\right)}^{4}\right]\|x_{🟉}{\|}^{4}+\frac{1}{2^{2d}}\left(\frac{3}{2}K_{2}d^{-44}+\frac{\pi d}{2}+272d^{4}\right)\sqrt{\epsilon }{\left\|x_{🟉}\right\|}^{4}+K_{2}\frac{\|x_{🟉}{\|}^{4}}{2^{2d}}d^{-44}. \end{array}$$

Similarly if $y\in B(-\phi_{d}\;x_{🟉},\varphi \|x_{🟉}\|)$, and φ=ϵ we obtain:

$$\begin{array}{cc} f_{H}\left(y\right) & \geq f_{E}\left(y\right)-|f_{0}\left(y\right)-f_{E}\left(y\right)|-\frac{1}{2}\left|\langle G\left(y\right)G{\left(y\right)}^{T}-G\left(x_{🟉}\right)G{\left(x_{🟉}\right)}^{T},H\rangle \right|\\ & \geq \frac{1}{2^{2d+2}}\left[1-2\phi_{d}^{2}\rho_{d}^{2}+{\left(\epsilon -\phi_{d}\right)}^{4}\right]\|x_{🟉}{\|}^{4}-\frac{1}{2^{2d}}\left(\frac{3}{2}2^{d}{\left\|x_{🟉}\right\|}^{-2}\omega +10\pi d^{3}+272d^{4}\right)\sqrt{\epsilon }\|x_{🟉}{\|}^{4}-\frac{\omega }{2^{d}}{\left\|x_{🟉}\right\|}^{2}\\ & \geq \frac{1}{2^{2d+2}}\left[1-2\phi_{d}^{2}\rho_{d}^{2}+{\left(\epsilon -\phi_{d}\right)}^{4}\right]\|x_{🟉}{\|}^{4}-\frac{1}{2^{2d}}\left(\frac{3}{2}K_{2}d^{-44}+10\pi d^{3}+272d^{4}\right)\sqrt{\epsilon }{\left\|x_{🟉}\right\|}^{4}-K_{2}\frac{\|x_{🟉}{\|}^{4}}{2^{2d}}d^{-44}. \end{array}$$

In order to guarantee that f(y)>f(x), it suffices to have:

$$2\left(1-\rho_{d}^{2}\right)\phi_{d}^{2}-8K_{2}d^{-44}>4C_{d}\sqrt{\epsilon }$$

with $C_{d}:=\left(544d^{4}+10\pi d^{3}\pi +3K_{2}d^{-44}+\pi d/2+1/100\right)$, that is to require:

$$\varphi =\epsilon <{\left(\frac{\left(1-\rho_{d}^{2}\right)\phi_{d}^{2}-4K_{2}d^{-44}}{2C_{d}}\right)}^{2}.$$

Finally notice that by Lemma 17 in [16] it holds that $1-\rho_{d}\geq {\left(K\left(d+2\right)\right)}^{-2}$ for some numerical constant *K*, we therefore choose $\phi =\varrho /d^{12}$ for some ϱ>0 small enough. □

### Appendix B.5. Supplementary Proofs for Appendix A.6

In this section we use strong convexity and smoothness to prove convergence to $x_{🟉}$ up to the noise variance ω. The idea is to show that every vector in the subgradient points in the direction $\left(x-x_{🟉}\right)$. Recall that the gradient in the noiseless case was:

$${\bar{v}}_{x}=\Lambda_{x}^{T}\left[\Lambda_{x}xx^{T}\Lambda_{x}^{T}-\Lambda_{x_{🟉}}x_{🟉}{x_{🟉}}^{T}\Lambda_{x_{🟉}}^{T}\right]\Lambda_{x}x.$$

We show that by continuity of $\Lambda_{x}$, when *x* is close to $x_{🟉}$, then ${\bar{v}}_{x}$ it is close to:

$${\bar{\bar{v}}}_{x}:=\Lambda_{x}^{T}\Lambda_{x}\left[xx^{T}-x_{🟉}{x_{🟉}}^{T}\right]\Lambda_{x}^{T}\Lambda_{x}x.$$

which in turn concentrates around:

$${\overset{ˇ}{v}}_{x}:=\frac{1}{2^{2d}}\left[xx^{T}-x_{🟉}{x_{🟉}}^{T}\right]x$$

by the WDC.

We begin by recalling the following result which can be found in the proof of Lemma 22 of [16].

**Lemma** **A16.**
*Suppose $x\in B(x_{🟉},d\sqrt{\epsilon }\|x_{🟉}\|)$ and the WDC holds with $\epsilon <1/(200^{4}d^{6})$. Then it holds that:*

$$∥\Lambda_{x}^{T}\Lambda_{x}x_{🟉}-\Lambda_{x}^{T}\Lambda_{x_{🟉}}x_{🟉}\|\leq \frac{1}{16}\frac{1}{2^{d}}\left\|x-x_{🟉}\right\|.$$

We now prove that ${\bar{v}}_{x}\approx {\bar{\bar{v}}}_{x}$ for *x* close to $x_{🟉}$.

**Lemma** **A17.**
*Suppose $x\in B(x_{🟉},d\sqrt{\epsilon }\|x_{🟉}\|)$ and the WDC holds with $\epsilon <1/(200^{4}d^{6})$. Then it holds that:*

$$∥{\bar{v}}_{x}-{\bar{\bar{v}}}_{x}\|\leq \frac{13}{96}\;\frac{1}{2^{2d}}\left\|x\|\right\|x_{🟉}\|\|x-x_{🟉}∥$$

**Proof.** Let $g_{x,🟉}:=\Lambda_{x}^{T}\Lambda_{x}x_{🟉}$ and $q_{x,🟉}:=\Lambda_{x}^{T}\Lambda_{x_{🟉}}x_{🟉}$. Then observe that:

$$\begin{array}{cc} \|{\bar{v}}_{x}-{\bar{\bar{v}}}_{x}\| & =\|\langle g_{x,🟉},x\rangle g_{x,🟉}-\langle q_{x,🟉},x\rangle q_{x,🟉}\|\\ & \leq \left(\|x\|\right\|g_{x,🟉}\left\|+\right\|\Lambda_{x}x\|\|\Lambda_{x_{🟉}}x_{🟉}\left\|)\right\|g_{x,🟉}-q_{x,🟉}∥\\ & \leq \frac{13}{6}\frac{1}{2^{d}}\left\|x\|\right\|x_{🟉}\left\|\right\|g_{x,🟉}-q_{x,🟉}∥\\ & \leq \frac{13}{96}\;\frac{1}{2^{2d}}\left\|x\|\right\|x_{🟉}\|\|x-x_{🟉}∥ \end{array}$$

where the second inequality follows from Lemma A1, and the third from Lemma A16. □

We next prove that by the WDC ${\bar{\bar{v}}}_{x}\approx {\overset{ˇ}{v}}_{x}$.

**Lemma** **A18.**
*Suppose the WDC holds with $\epsilon <1/{\left(16\pi d^{2}\right)}^{2}$. Then for all nonzero $x,x_{🟉}$:*

$$∥{\bar{\bar{v}}}_{x}-{\overset{ˇ}{v}}_{x}\|\leq \frac{25}{12}\;\frac{4\epsilon d}{2^{2d}}\|x\|\|x-x_{🟉}\|\|x+x_{🟉}∥$$

**Proof.** For notational convenience we define $E_{d}:=I_{k}/2^{d}$ the scaled identity in $R^{d}$, $Q_{x}:=\Lambda_{x}^{T}\Lambda_{x}$ and $M_{x,🟉}=xx^{T}-x_{🟉}{x_{🟉}}^{T}$. Next observe that:

$$\begin{array}{cc} \|{\bar{\bar{v}}}_{x}-{\overset{ˇ}{v}}_{x}\| & \leq \|\left(Q_{x}-E_{d}\right)M_{x,🟉}Q_{x}x\|+\|E_{d}M_{x,🟉}\left(Q_{x}-E_{d}\right)x\|,\\ & \leq \frac{25}{12}\;\frac{1}{2^{d}}\|Q_{x}-E_{d}\left\|\right\|M_{x,🟉}\left\|x\|\right|\\ & \leq \frac{25}{12}\;\frac{4\epsilon d}{2^{2d}}\|M_{x,🟉}\left\|\|x\right\| \end{array}$$

where the second inequality follows from Lemma A1 and the third from (17) in [15]. We conclude by noticing that $\|M_{x,🟉}\|\leq \|x-x_{🟉}\|\|x+x_{🟉}\|$. □

Next consider the quartic function $\overset{ˇ}{f}\left(x\right):=1/2^{2d+2}{\left\|xx^{T}-x_{🟉}{x_{🟉}}^{T}\right\|}_{F}^{2}$ and observe that:

$$\begin{array}{cc} \nabla \overset{ˇ}{f}\left(x\right) & =\frac{1}{2^{2d}}\left(xx^{T}-x_{🟉}{x_{🟉}}^{T}\right)x={\overset{ˇ}{v}}_{x},\\ \nabla^{2}\overset{ˇ}{f}\left(x\right) & =\frac{1}{2^{2d}}{\left(\|x\right\|}^{2}I_{n}+2xx^{T}-x_{🟉}{x_{🟉}}^{T}). \end{array}$$

Following [63] we show that ${\overset{ˇ}{v}}_{x}$ is β-smooth and strongly convex, in turn deriving the result in Lemma A19.

**Lemma** **A19.**
*Assume $\|x-x_{🟉}\left\|\leq \gamma \right\|x_{🟉}\|$ with γ<1/5. Take $\tau \geq 3{\left(1+\gamma \right)}^{2}+1$, then:*

$$∥{\overset{ˇ}{v}}_{x}-\frac{\tau }{2^{2d}}\|x_{🟉}{\|}^{2}\left(x-x_{🟉}\right)\|\leq \sqrt{\tau (\tau -\alpha )}\left\|x-x_{🟉}\right\|\frac{\|x_{🟉}{\|}^{2}}{2^{2d}}$$

*where α=2−9γ.*

**Proof.** Let $\|x-x_{🟉}\left\|\leq \gamma \right\|x_{🟉}\|$ with γ<1/5, then:

(A39) $$\begin{array}{c} \left(1-\gamma \right)\|x_{🟉}\left\|\leq \|x\|\leq \left(1+\gamma \right)\right\|x_{🟉}\| \end{array}$$

(A40) $$\begin{array}{c} \left\|\Delta \|\|x\|\leq \gamma \left(1+\gamma \right)\right\|x_{🟉}{\|}^{2} \end{array}$$

since $\Delta =x-x_{🟉}$ satisfies ‖Δ‖≤‖x‖. Using (A40) we then obtain

$$\begin{array}{cc} xx^{T} & =x_{🟉}{x_{🟉}}^{T}+\Delta x^{T}+x\Delta^{T}-\Delta \Delta^{T},\\ & ⪰x_{🟉}{x_{🟉}}^{T}-3\|\Delta \|\|x\|I_{k},\\ & ⪰x_{🟉}{x_{🟉}}^{T}-3\gamma \left(1+\gamma \right){\left\|x_{🟉}\right\|}^{2}I_{k}. \end{array}$$

We therefore have:

$$\begin{array}{cc} 2^{2d}\nabla^{2}\overset{ˇ}{f}\left(x\right) & ={\left\|x\right\|}^{2}I_{k}+2xx^{T}-x_{🟉}{x_{🟉}}^{T}\\ & ⪰{\left\|x\right\|}^{2}I_{k}+x_{🟉}{x_{🟉}}^{T}-6\gamma \left(1+\gamma \right){\left\|x_{🟉}\right\|}^{2}I_{k},\\ & ⪰\left({\left(1-\gamma \right)}^{2}+1-6\gamma \left(1+\gamma \right)\right){\left\|x_{🟉}\right\|}^{2}I_{k},\\ & ⪰\left(2-9\gamma \right)\|x_{🟉}{\|}^{2}I_{k}, \end{array}$$

where in the second line we have used (A39) and in the third 0<γ<1/5. Next notice that using (A39) we have

$$2^{2d}\|\nabla^{2}\overset{ˇ}{f}{\left(x\right)\|\leq 3\|x\|}^{2}+\|x_{🟉}{\|}^{2}\leq \left(3{\left(1+\gamma \right)}^{2}+1\right){\left\|x_{🟉}\right\|}^{2}$$

and therefore, for all $x\in B(x_{🟉},\gamma \|x_{🟉}\|)$

$$\left(2-9\gamma \right)\|x_{🟉}{\|}^{2}I_{k}⪯2^{2d}\nabla^{2}\overset{ˇ}{f}\left(x\right)⪯\left(3{\left(1+\gamma \right)}^{2}+1\right){\left\|x_{🟉}\right\|}^{2}.$$

The above bounds imply in particular that for all $x\in B(x_{🟉},\gamma \|x_{🟉}{\|}^{2})$ the gradient of $\overset{ˇ}{f}$ satisfies the *regularity condition* (see [63]):

(A41) $$2\langle \nabla \overset{ˇ}{f}\left(x\right),x-x_{🟉}\rangle \geq \mu \|\nabla \overset{ˇ}{f}{\left(x\right)\|}^{2}+\lambda {\left\|x-x_{🟉}\right\|}^{2},\;$$

where $\lambda =\left(2-9\gamma \right)\|x_{🟉}{\|}^{2}/2^{2d}$ and $1/\mu =\left(3{\left(1+\gamma \right)}^{2}+1\right){\left\|x_{🟉}\right\|}^{2}/2^{2d}$. By (A41) we can then conclude that for σ≥1/μ:

$$\begin{array}{cc} \|\nabla \overset{ˇ}{f}\left(x\right)-\sigma \left(x-x_{🟉}\right){\|}^{2} & \leq \|\nabla \overset{ˇ}{f}{\left(x\right)\|}^{2}+\sigma^{2}{\left\|x-x_{🟉}\right\|}^{2}-2\sigma \langle \nabla \overset{ˇ}{f}\left(x\right),x-x_{🟉}\rangle \\ & \leq \left(1-\sigma \mu \right)\|\nabla \overset{ˇ}{f}{\left(x\right)\|}^{2}+\sigma \left(\sigma -\lambda \right){\left\|x-x_{🟉}\right\|}^{2}\\ & \leq \sigma \left(\sigma -\lambda \right)\|x-x_{🟉}{\|}^{2} \end{array}$$

Finally letting $\sigma =\tau \|x_{🟉}{\|}^{2}/2^{d}$, α=(2−9γ), and by the assumptions on γ and τ the thesis follows. □

We finally can prove Lemma A9.

**Proof of Lemma A9.** Let $x\in B(x_{🟉},d\sqrt{\epsilon }\|x_{🟉}\|)$, then $\|x+x_{🟉}\|\leq \left(2+d\sqrt{\epsilon }\right)\left\|x_{🟉}\right\|$ and since $\epsilon <1/(200^{4}d^{6})$ we have from Lemma A17 and Lemma A18

$$∥{\bar{v}}_{x}-{\overset{ˇ}{v}}_{x}\left\|\leq \right\|{\bar{v}}_{x}-{\bar{\bar{v}}}_{x}\left\|+\right\|{\bar{\bar{v}}}_{x}-{\overset{ˇ}{v}}_{x}\|\leq \frac{1}{2^{2d}}\frac{13}{48}\left\|x\|\right\|x_{🟉}\|\|x-x_{🟉}\|.$$

If $x\in B(x_{🟉},d\sqrt{\epsilon }\|x_{🟉}\|)$ is a differentiable point of *f*, then by Lemma A19 and the assumption (13) on the noise *H*, it holds that:

$$∥{\tilde{v}}_{x}-\frac{\tau }{2^{2d}}\|x_{🟉}{\|}^{2}\left(x-x_{🟉}\right)\|\leq \left[\frac{13}{48}\left(1+\gamma \right)+\sqrt{\tau (\tau -\alpha )}\right]\frac{\|x_{🟉}{\|}^{2}}{2^{2d}}\|x-x_{🟉}\|+\frac{\omega }{2^{d}}\left(1+\gamma \right)\left\|x_{🟉}\right\|.$$

where $\gamma =d\sqrt{\epsilon }$, α=2−9γ and $\tau \geq 3{\left(1+\gamma \right)}^{2}+1$. In general, if $x\in B(x_{🟉},\gamma \|x_{🟉}\|)$ and $v_{x}\in \partial f\left(x\right)$, by the characterization of Clarke subdifferential (9) and the previous results:

$$\begin{array}{cc} ∥v_{x}-\frac{\tau }{2^{2d}}\|x_{🟉}{\|}^{2}\left(x-x_{🟉}\right)\| & \leq \sum_{t=1}^{T}c_{t}\|v_{t}-\frac{\tau }{2^{2d}}\|x_{🟉}{\|}^{2}\left(x-x_{🟉}\right)\|\\ & \leq \left[\frac{13}{48}\left(1+\gamma \right)+\sqrt{\tau (\tau -\alpha )}\right]\frac{\|x_{🟉}{\|}^{2}}{2^{2d}}\|x-x_{🟉}\|+\frac{\omega }{2^{d}}\left(1+\gamma \right)\left\|x_{🟉}\right\|. \end{array}$$

We finally obtain the thesis by noting that by the assumptions on ϵ it holds that $\gamma =d\sqrt{\epsilon }<1/{\left(400\right)}^{2}$ and taking $\tau =\tau_{1}=21/5$, $\tau_{2}=17/5$ and $\tau_{3}=\left(1+1/{\left(400\right)}^{2}\right)$. □

## Appendix C. Proof of Theorem 1

By Theorem 3 it suffices to show that with high probability the weight matrices ${\left\{W_{i}\right\}}_{i=1}^{d}$ satisfy the WDC, and that $\omega /2^{d}$ upper bounds the spectral norm of the noise term $\Lambda_{x}^{T}H\Lambda_{x}$ where

- in the **Spiked Wishart Model** $H=\Sigma_{N}-\Sigma$ where $\Sigma_{N}=Y^{T}Y/N$ and the $\Sigma =y_{🟉}{y_{🟉}}^{T}+\sigma^{2}I_{n}$;
- in the **Spiked Wigner Model** H=νH where H∼GOE(n).

Regarding the Weight Distribution Condition (WDC), we observe that it was initially proposed in [15], where it was shown to hold with high probability for networks with random Gaussian weights under an expansivity condition on their dimensions. It was later shown in [62] that a less restrictive expansivity rate is sufficient.

**Lemma** **A20** (Theorem 3.2 in [62]). *There are constants C,c>0 with the following property. Let 0<ϵ<1 and suppose $W\in R^{n\times k}$ has i.i.d.N(0,1/n) entries. Suppose that $n\geq c\;k\;\epsilon^{-2}\;\log\left(1/\epsilon \right)$. Then with probability at least 1−exp(−Ck), W satisfies the WDC with constant ϵ.*

By a union bound over all layers, using the above result we can conclude that the WDC holds simultaneously for all layers of the network with probability at least $1-\sum_{i=1}^{d}e^{-Cn_{i-1}}.$ Note in particular that this argument does not requires the independence of the weight matrices ${\left\{W_{i}\right\}}_{i=1}^{d}$.

By Lemma A20, with high probability the random generative network *G* satisfies the WDC. Therefore if we can guarantee that the assumptions on the noise term *H* are satisfied, then the proof of the main Theorem 1 follows from the deterministic Theorem 3 and Lemma A20.

Before turning to the bounds of the noise terms in the spiked models, we recall the following lemma which bounds the number of possible $\Lambda_{x}$ for x≠0. Note that this is related to the number of possible regions defined by a deep ReLU network.

**Lemma** **A21.**
*Consider a network G as defined in (3) with d≥2, weight matrices $W_{i}\in R^{n_{i}\times n_{i-1}}$ with i.i.d. entries $N(0,1/n_{i})$. Then, with probability one, for any x≠0 the number of different matrices $\Lambda_{x}$ is*

$$|\left\{\Lambda_{x}|x\neq 0\right\}|\leq 10^{d^{2}}{\left(n_{1}^{d}n_{2}^{d-1}\cdots n_{d}\right)}^{k}\leq {\left(n_{1}^{d}n_{2}^{d-1}\cdots n_{d}\right)}^{8k}$$

**Proof.** The first inequality follows from Lemma 16 and the proof of Lemma 17 in [15]. For the second inequality notice that as k≥1, $n_{1}>k$ and d≥2 it follows that $7k\log\left(n_{1}^{d}n_{2}^{d-1}\cdots n_{d}\right)\geq 7kd(d+1)\log\left(n_{1}\right)/2\geq 3.5d\left(d+1\right)\log\left(2\right)\geq d^{2}\log\left(10\right).$ □

In the next section we use this lemma to control the noise term $\Lambda_{x}^{T}H\Lambda_{x}$ on the event where the WDC holds.

### Appendix C.1. Spiked Wigner Model

Recall that in the Wigner model $Y=G\left(x_{🟉}\right)G{\left(x_{🟉}\right)}^{T}+\nu H$ and the symmetric noise matrix H follows a *Gaussian Orthogonal Ensemble* GOE(n), that is $H_{ii}∼N\left(0,2/n\right)$ for all 1≤i≤n and $H_{ji}=H_{ij}∼N\left(0,1/n\right)$ for 1≤j<i≤n. Our goal is to bound $\|\Lambda_{x}^{T}H\Lambda_{x}\|$ uniformly over *x* with high probability.

Fix $x\in R^{k}$, and let $N_{1/4}$ be a 1/4-net on the sphere $S^{k-1}$ such that (see for example [64]) $|N_{1/4}|\leq 9^{k}$ and

$$∥\Lambda_{x}^{T}H\Lambda_{x}\|\leq 2\max_{z\in N_{1/4}}\left|\langle \Lambda_{x}^{T}H\Lambda_{x}z,z\rangle \right|.$$

Observe next that for any $v\in R^{n}$ by the definition of GOE(n) it holds that

$$v^{T}Hv=\sum_{i}^{n}H_{ii}v_{i}^{2}+2\sum_{i<j}^{n}H_{ij}v_{i}v_{j}∼N(0,2(\sum_{i}v_{i}^{4}+2\sum_{i<j}v_{i}^{2}v_{j}^{2})/n)=N({0,2\|v\|}^{4}/n).$$

Therefore for any $z\in N_{1/4}$ let $\ell_{x,z}:=\Lambda_{x}z\in R^{n}$, then $\ell_{x,z}^{T}H\ell_{x,z}∼N\left(0,2\|\ell_{x,z}{\|}^{4}/n\right)$. In particular by (A6), the quadratic form $\ell_{x,z}^{T}H\ell_{x,z}$ is sub-Gaussian with parameter $\gamma^{2}$ given by

$$\gamma^{2}:=\frac{2}{n}{\left(\frac{13}{12}\right)}^{2}\frac{1}{2^{2d}}.$$

Then for fixed $x\in R^{k}$, standard sub-Gaussian tail bounds (e.g., [64]) and a union bound over $N_{1/4}$ give for any u≥0

$$\begin{array}{cc} P\left[\|\Lambda_{x}^{T}H\Lambda_{x}\|\geq 2u\right] & \leq P[\max_{z\in N_{1/4}}\left\|\ell_{x,z}^{T}H\ell_{x,z}\right\|\geq u]\\ & \leq \sum_{z\in N_{1/4}}P\left[\|\ell_{x,z}^{T}H\ell_{x,z}\|\geq u\right]\leq 2\cdot 9^{k}e^{-\frac{u^{2}}{2\gamma^{2}}}. \end{array}$$

Lemma A21, then ensures that the number of possible $\Lambda_{x}$ is at most ${\left(n_{1}^{d}n_{2}^{d-1}\cdots n_{d}\right)}^{8k}$, so a union bound over this set allows us to conclude that

$$\begin{array}{cc} P\left[\|\Lambda_{x}^{T}H\Lambda_{x}\|\leq 2u,\;\mathrm{for}\;\mathrm{all}\;x\right] & \geq 1-{\left(n_{1}^{d}n_{2}^{d-1}\cdots n_{d}\right)}^{8k}P\left[\|\Lambda_{x}^{T}H\Lambda_{x}\|\geq 2u\right]\\ & \geq 1-2\exp\left(8k\log\left(2\;n_{1}^{d}n_{2}^{d-1}\cdots n_{d}\right)-u^{2}/\left(2\gamma^{2}\right)\right) \end{array}$$

Choosing then $u=\sqrt{2\gamma^{2}\cdot 9k\log\left(2\;n_{1}^{d}n_{2}^{d-1}\cdots n_{d}\right)}$ and substituting the definition of $\gamma^{2}$, we obtain

$$P[\|\Lambda_{x}^{T}H\Lambda_{x}\|\leq \frac{1}{2^{d}}\sqrt{\frac{169k\log(2\;n_{1}^{d}n_{2}^{d-1}\cdots n_{d})}{n}},\;\mathrm{for}\;\mathrm{all}\;x]\geq 1-2e^{-k\log(2\;n_{1}^{d}n_{2}^{d-1}\cdots n_{d})}$$

which implies the thesis as $n=n_{d}$ and $\log\left(n\right)\leq \log\left(2\;n_{1}^{d}n_{2}^{d-1}\cdots n_{d}\right)$.

### Appendix C.2. Spiked Wishart Model

Each row ${\left\{y_{i}\right\}}_{i=1}^{N}$ of the matrix *Y* in (1) can be seen as i.i.d. samples from N(0,Σ) where $\Sigma =y_{🟉}{y_{🟉}}^{T}+\sigma^{2}I_{n}$. In the minimization problem (4) we take $M=\Sigma_{N}-\sigma^{2}I_{n}$ where $\Sigma_{N}$ is the empirical covariance matrix $Y^{T}Y/N$. The symmetric noise matrix *H* is then given by $H=\Sigma_{N}-\Sigma$ and by the Law of Large Numbers H→0 as N→∞. We bound $\|\Lambda_{x}^{T}H\Lambda_{x}\|$ with high probability uniformly over $x\in R^{k}$.

Fix $x\in R^{k}$, let $N_{1/4}$ be a 1/4-net on the sphere $S^{k-1}$ such that $|N_{1/4}|\leq 9^{k}$, and notice that:

$$\|\Lambda_{x}^{T}H\Lambda_{x}\|\leq 2\max_{z\in N_{1/4}}\left|z^{T}\Lambda_{x}^{T}H\Lambda_{x}z\right|.$$

By a union bound on $N_{1/4}$ we obtain for any fixed $z\in N_{1/4}$:

$$P\left[\|\Lambda_{x}^{T}H\Lambda_{x}\|\geq 2u\right]\leq 9^{k}P\left[|z^{T}\Lambda_{x}^{T}H\Lambda_{x}z|\geq u\right].$$

Let $\ell_{x,z}:=\Lambda_{x}z$ and $s_{i}:=\ell_{x,z}^{T}y_{i}$, so that we can write

$$\begin{array}{cc} z^{T}\Lambda_{x}^{T}H\Lambda_{x}z & =\frac{1}{N}\sum_{i=1}^{N}\left({\left(\ell_{x}^{T}y_{i}\right)}^{2}-E\left[{\left(\ell_{x}^{T}y_{i}\right)}^{2}\right]\right)=\frac{1}{N}\sum_{i=1}^{N}\left(s_{i}^{2}-E\left[s_{i}^{2}\right]\right) \end{array}$$

and in particular

$$P\left[\|\Lambda_{x}^{T}H\Lambda_{x}\|\geq 2u\right]\leq 9^{k}P\left[|z^{T}\Lambda_{x}^{T}H\Lambda_{x}z|\geq u\right]=9^{k}P\left[\frac{1}{N}\left|\sum_{i=1}^{N}s_{i}^{2}-E\left[s_{i}^{2}\right]\right|\geq u\right]$$

Observe then that $s_{i}∼N\left(0,\gamma^{2}\right)$ with $\gamma^{2}=\ell_{x,z}^{T}\Sigma \ell_{x,z}$. It follows for $u\in [0,\gamma^{2}]$ by the small deviation bound for $\chi^{2}$ random variables (e.g., [33] (Example 2.11))

$$P\left[\|\Lambda_{x}^{T}H\Lambda_{x}\|\geq 2u\right]\leq 2\;\exp\left(2k\log3-\frac{Nu^{2}}{8\gamma^{4}}\right)$$

Recall now that $|\left\{\Lambda_{x}|x\neq 0\right\}|\leq {\left(n_{1}^{d}n_{2}^{d}\cdots n_{d}\right)}^{8k}$, then proceeding as for the Wigner case by a union bound over all possible $\Lambda_{x}$:

$$\begin{array}{cc} P\left[\|\Lambda_{x}^{T}H\Lambda_{x}\|\leq 2u,\;\mathrm{for}\;\mathrm{all}\;x\right] & \geq 1-{\left(n_{1}^{d}n_{2}^{d-1}\cdots n_{d}\right)}^{8k}P\left[\|\Lambda_{x}^{T}H\Lambda_{x}\|\geq 2u\right]\\ & \geq 1-2\exp(8k\log\left(2\;n_{1}^{d}n_{2}^{d-1}\cdots n_{d}\right)-\frac{Nu^{2}}{8\gamma^{4}}). \end{array}$$

Substituting $u=\sqrt{8\gamma^{4}\cdot 9k\log\left(2\;n_{1}^{d}n_{2}^{d-1}\cdots n_{d}\right)/N}$ we find that:

(A42) $$P[\|\Lambda_{x}^{T}H\Lambda_{x}\|\leq 2\sqrt{\frac{72k\log(2\;n_{1}^{d}n_{2}^{d-1}\cdots n_{d})}{N}}\gamma^{2},\;\mathrm{for}\;\mathrm{all}\;x]\geq 1-2e^{-k\log(n)}$$

since $\log\left(n\right)\leq \log\left(2\;n_{1}^{d}n_{2}^{d-1}\cdots n_{d}\right)$.

Similarly if $u>\gamma^{2}$ by large deviation bounds for sub-exponential variables

$$P\left[\|\Lambda_{x}^{T}H\Lambda_{x}\|\leq 2u,\;\mathrm{for}\;\mathrm{all}\;x\right]\geq 1-2\exp(8k\log\left(2\;n_{1}^{d}n_{2}^{d-1}\cdots n_{d}\right)-\frac{Nu}{8\gamma^{2}}).$$

Substituting $u=8\gamma^{2}\cdot 9k\log\left(2\;n_{1}^{d}n_{2}^{d-1}\cdots n_{d}\right)/N$ we find that:

(A43) $$P[\|\Lambda_{x}^{T}H\Lambda_{x}\|\leq 2\frac{72k\log(3\;n_{1}^{d}n_{2}^{d-1}\cdots n_{d})}{N}\gamma^{2},\;\mathrm{for}\;\mathrm{all}\;x]\geq 1-2e^{-k\log(n)}$$

Finally observe that using (A6) for bounding $\|\Lambda_{x}{\|}^{2}$ (by the WDC) and $\sigma^{2}+{\left\|y_{🟉}\right\|}^{2}$ for bounding ‖Σ‖, we have

$$\gamma^{2}\leq \frac{13}{12}\frac{1}{2^{d}}\left(\right\|y_{🟉}{\|}^{2}+\sigma^{2}),$$

which combined with (A42) and (A43) implies the thesis.
